# Farrerol Ameliorated Cisplatin-Induced Chronic Kidney Disease Through Mitophagy Induction *via* Nrf2/PINK1 Pathway

**DOI:** 10.3389/fphar.2021.768700

**Published:** 2021-11-11

**Authors:** Ning Ma, Zhentong wei, Jianqiang Hu, Wenjing Gu, Xinxin Ci

**Affiliations:** ^1^ Institute of Translational Medicine, The First Hospital of Jilin University, Changchun, China; ^2^ Department of Obstetrics and Gynecology, The First Hospital of Jilin University, Changchun, China; ^3^ Department of Otolaryngology Head and Neck Surgery, The First Hospital of Jilin University, Changchun, China

**Keywords:** chronic kidney disease, mitophagy, Nrf2, PINK1, acute kidney injury

## Abstract

Previously, Our study has showed that farrerol can activate Nrf2 and ameliorate cisplatin-induced acute kidney injury (AKI). Mitophagy reportedly can prevent diabetic nephropathy, cisplatin-induced AKI and other related nephropathy. In this study, we evaluated the correlation between mitophagy and the protective effect of the Nrf2 activator farrerol on cisplatin-induced CKD by using C57BL/6 wild-type and Nrf2 knockout mice. We confirmed that Nrf2 and PINK1/Parkin-mediated mitophagy was significantly increased on the 3rd day of cisplatin stimulation but was reduced on the 38th day of cisplatin stimulation. Similar to previous results, farrerol activated Nrf2 on the 38th day of cisplatin administration, subsequently stimulating the Nrf2-targeted antioxidant enzymes HO-1 and NQO1. In addition, farrerol triggered PINK1/Parkin-mediated mitophagy by recruiting the receptor proteins LC3 and p62/SQSTM1, thereby eliminating damaged mitochondria. Furthermore, genetic deletion of Nrf2 reduced PINK1/Parkin-mediated mitophagy activation and led to increased renal tubular necrosis and renal fibrosis. We also found that farrerol alleviated inflammation and renal fibrosis by inhibiting p-NF-κB/NLRP3 and TGF-*β*/Smad signaling. These data indicated that farrerol effectively inhibited cisplatin-induced inflammation and renal fibrosis by activating Nrf2 and PINK1/Parkin-mediated mitophagy, which provides a potential novel therapeutic target for CKD.

## Introduction

Cisplatin (CDDP), a platinum drug, has been universally utilized to treat bladder cancer, ovarian cancer and other solid tumors (Dasari and Tchounwou, 2014). However, the dominant factor restricting the clinical use of cisplatin is its nephrotoxicity (Zhu et al., 2015). Although hydration is extensively used to eliminate cisplatin-induced nephrotoxicity in clinical practice, patients receiving cisplatin chemotherapy remain at a higher hazard of acute kidney injury (AKI) (Pabla and Dong, 2008). Moreover, patients with severe and recurrent AKI are more likely to develop chronic kidney disease (CKD), which is accompanied by persistent renal dysfunction, development of fibrosis, and inflammation (Chawla et al., 2011; Thakar et al., 2011; Basile et al., 2016). What’s more, the death rate of CKD has continued to increase at a rate of 1% annually, and this life-threatening disease has become a global burden in the past few years (Eckardt et al., 2013). Owing to the lack of knowledge about the pathological mechanisms involved in the development of CKD, there are currently few clinical strategies or treatments that substantially improve kidney function or prevent disease progression (Bin et al., 2017; Forbes and Thorburn, 2018). Therefore, there is an urgent need to study and understand the mechanism of CKD occurrence and development.

Cell necrosis and inflammation in the proximal tubules are hallmarks of cisplatin-induced AKI (CDDP-AKI), and maladaptive or incomplete repair of kidney tubules following serious or recurrent AKI causes kidney fibrosis and ultimately exacerbates CKD (Himmelfarb et al., 2002; Vaziri, 2004). When a large amount of cisplatin accumulates in epithelial tubule cells, it can induce cells to produce excessive reactive oxygen species (ROS) due to mitochondrial dysfunction, leading to cisplatin-induced renal dysfunction (Szeto, 2006; Oh et al., 2016; Wei et al., 2020). Excessive production of ROS in cells disrupts the redox balance in CKD, causing further oxidative stress, kidney tissue damage and dysfunction (Ma et al., 2019).

Moreover, damaged renal tubular epithelial cells can induce inflammation by triggering a variety of proinflammatory cytokines, during which NF-κB, an important regulator of the renal inflammatory response mechanism, activates the expression of NLRP3 (Lorenz et al., 2014; Guo et al., 2015). Subsequently, the NLRP3 inflammasome triggers cleaved caspase-1 and IL-1*β* activation, which in turn triggers the release of proinflammatory mediators (Oberg et al., 2004; Tucker et al., 2015; Xu et al., 2015). In addition, persistent kidney damage or chronic unresolved inflammation can cause tissue repair failure and promote the production of tumor growth factor *β* (TGF-*β*) (Meng et al., 2015; Qin et al., 2016). A growing number of studies have illustrated that under pathological stimulation, overexpression of TGF-*β* in mouse kidneys can directly stimulate the expression of excessive collagen I and alpha smooth muscle actin (*α*-SMA) proteins, by activating regulatory Smad transcription factors (Liu, 2006; Meng et al., 2015). In addition, TGF-*β* increases the level of ROS in the kidney, and the generated ROS stimulate TGF-*β*-related fibroblast activation and myofibroblast differentiation, which further promotes the development of renal fibrosis (Kopp et al., 1996; Clouthier et al., 1997).

Nuclear factor erythrocyte 2-related factor 2 (Nrf2) is stimulated by ROS-mediated tissue damage, liberated from Keap1 and transferred to the nucleus. Subsequently, Nrf2 combines with small Maf (sMaf) proteins to form heterodimers that induce the expression of downstream antioxidants and detoxification enzymes, including heme oxygenase-1 (HO-1), NAD(P)H quinone oxidoreductase 1 (NQO1), superoxide dismutase (SOD) and glutathione (GSH) (Qin et al., 2016; Wei et al., 2020). To a certain extent, Nrf2 balances the effect of hydrogen peroxide and lipid peroxidation, thus ameliorating TGF-*β*-mediated profibrotic signals (Oh et al., 2012a; Oh et al., 2012b). Moreover, one study pointed out that Nrf2-mediated PINK1 transcriptional regulation restores mitophagy and abnormal mitochondrial dynamics in renal tubular cells (Xiao et al., 2017). More experiments have shown that autophagy alleviates protein aggregates in the endoplasmic reticulum and mitochondria as well as other specific cargoes with high selectivity (Mizumura et al., 2014; Guimaraes et al., 2015; Yoshii and Mizushima, 2015). Among these processes, mitophagy specifically eliminates excessive and/or damaged mitochondria (Khaminets et al., 2015). Due to the abundant mitochondria and higher rate of oxygen consumption, mitophagy is particularly necessary to maintaining the homeostasis of mitochondria in the kidneys (Ralto et al., 2020). PINK1/Parkin-mediated mitophagy is the most important mechanism for identifying and labeling mitochondria under cellular stress. Ubiquitinated PINK1/Parkin recruits the receptor protein p62/SQSTM1, which causes autophagosome formation and elimination of damaged mitochondria by connecting ubiquitin-labeled mitochondria with LC3 in the autophagosome membrane (Bueno et al., 2015; Zimmermann and Reichert, 2017). Previous studies have found that knocking down PINK1/Parkin enhances cisplatin-induced mitochondrial dysfunction and increases human renal proximal tubular cell damage by inhibiting mitophagy (Zhao et al., 2017). In addition, diabetic mouse kidney tubular damage is partially reversed and mitochondrial fragmentation and apoptosis are improved by regulating the Nrf2/PINK1-mediated mitophagy of renal tubular cells (Xiao et al., 2017). Moreover, new strategies for Nrf2/PINK1-mediated mitophagy have been used to treat kidney disease in CKD animal models. Many natural products that activate Nrf2 can counteract oxidative damage by controlling the Nrf2/ARE signaling pathway.

Our previous experiments have proven that farrerol is a novel Nrf2 activator that can improve cisplatin-induced nephrotoxicity by activating Nrf2, thereby regulating the related oxidation, inflammation and apoptosis signaling pathways (Ma et al., 2019). The protective effect of farrerol against cisplatin-induced CKD (CDDP-CKD) has not been previously reported. Here, the function and underlying mechanism of farrerol in CDDP-CKD were measured and evaluated using related experimental models.

## Materials and Methods

### Reagents and Chemicals

Farrerol and cisplatin (15663-27-1) were obtained from Chengdu Pufei De Biotech Co., Ltd. (Chengdu, China) and MedChemExpress (New Jersey, United States), respectively. Primary antibodies against Nrf2(ab31163), Keap1 (ab139729), HO-1 (ab68477), NQO1 (ab80588), PINK1 (p0076), NOX4 (A11274) and *β*-actin (km9001) were obtained from Sigma-Aldrich (St. Louis, MO, United States), Sungene Biotech Co., Ltd (Tianjin, China) and Abcam (Cambridge, MA, United States). Antibodies specific to KIM-1 (AF 1817), NGAL (AF 1857), TGF-*β* (E-AB-33090), E-cadherin (E-AB-70249), Smad (E-AB-21040), collagen I (E-AB-34264), *α*-SMA (E-AB-21040), NLRP3 (MAB7578) and p-NF-Κb (13346) were purchased from R&D Systems (Minnesota, MN, United States), Elabscience Biotechnology (Wuhan, China) and Cell Signaling Technology (Boston, MA, United States).

### Experimental Design and Animal Procedures

C57BL/6 wildtype and Nrf2-knockout mice were purchased from Vital River (Beijing, China) and Jackson Laboratory (Bar Harbor, ME, United States), respectively. C57BL/6 wild-type and Nrf2 knockout mice were randomly assigned to experimental groups receiving the following treatments: control (saline), CDDP (10 mg/kg) only, farrerol (10 mg/kg) only, or CDDP (10 mg/kg) + farrerol (10 mg/kg). The mice were administered farrerol at 10 mg/kg body weight by intraperitoneal injection beginning 1 day before the first CDDP injection and daily until the day of harvest in the CDDP-AKI mouse group or 5 days after the second cisplatin injection in the CDDP-CKD mouse group. The mice were euthanized on day 3 (CDDP-AKI) or day 38 (CDDP-CKD) after the first administration of cisplatin. C57BL/6 wild-type and Nrf2 knockout mice were maintained on a normal diet and provided free access to drinking water during this experiment. All mice were kept in a specific pathogen-free facility, and this experiment was approved by the Animal Health and Research Ethics Committee of Jilin University.

### Biochemical Index Assays

Kidney function was analyzed according to the BUN and SCr levels. Serum samples of BUN and SCr were collected and measured using the relevant kits purchased from Nanjing Jiancheng Bioengineering Institute (Nanjing, China), and then the content of BUN (640 nm) and SCr (546 nm) in the serum sample is obtained by measuring the OD value and data processing. In addition, kidneys were homogenized and dissolved in extraction buffer, and the levels of MPO, MDA, SOD, and GSH were analyzed using kits (Keygen Biotech. Co., Ltd., Nanjing, China) according to the manufacturer’s instructions.

### Histological Analyses

Fresh kidney tissue was dissected and immediately fixed with 4% paraformaldehyde. Then, a microscope was used to analyze sections from the paraffin-embedded mouse kidneys stained with hematoxylin and eosin (H&E) or Masson reagents. The damaged tubule scores were divided into the following levels: grade 0, no damage; grade 1, <25%; grade 2, 25–49%; grade 3, 50–74%; and grade 4, ≥75%. Additionally, Masson’s staining was used to evaluate the renal tissue fibrosis area.

### Western Blotting

The kidney tissue protein was separated by 10 or 12.5% sodium lauryl sulfate polyacrylamide gel electrophoresis and transferred to a polyvinylidene fluoride membrane. The membrane was blocked and shaken in 5% skim milk and then incubated with the corresponding primary and secondary antibodies for protein detection. Then, we used ECL to observe the bands and utilized ImageJ gel analysis software to quantitatively analyze the band intensities.

### Immunohistochemistry

Paraffin-embedded kidney sections were deparaffinized and rehydrated. Then, we processed and microwaved the slices in citrate buffer and subsequently blocked them with 5% BSA for 20 min. The slides were incubated with primary antibodies against *α*-SMA and then incubated with secondary antibodies. Then, an optical microscope was used to observe the changes in kidney morphology and the area of positive staining.

### Transmission Electron Microscopy (TEM)

Fresh kidneys were harvested and prefixed with glutaraldehyde and then fixed with osmium tetroxide. Afterwards, the samples were dehydrated in ethanol containing 3% uranyl acetate and embedded in epoxy resin and propylene oxide. After polymerization, the samples were cut into 70 nm-thick sections, stained, and then inspected with TEM. The quantification of mitochondrial contour measurements (feret minimum and maximum, area, perimeter, aspect ratio, shape factor, and roundness) in the kidney tissue sample were calculated using ImageJ software. Feret minimum and maximum represent the longest and shorted diameter in mitochondrial. Area, perimeter, aspect ratio, and roundness describe the degree of flatness of a contour. The shape factor corresponds to the outline of a contours. Contour measures are related as follows:

$$Aspect\;Ratio\;=\;\frac{MaxDiameter}{MinDiameter}$$

$$Shape\;factor\;=\;\frac{Perimeter}{\sqrt{Area}}$$

$$Roundness\;=\;\frac{4\cdot Area}{\pi \cdot MaxDiameter^{2}}$$

### Cell Experiment

Human renal tubular epithelial cells line (HK-2) obtained from Chinese Cell Bank (Beijing, China) were maintained in DMEF containing 10% fetal bovine serum, 100U/ml penicillin-streptomycin, in a 37°C, 5% CO2 incubator. After 72 h of transfection of HK-2 cells with the control siRNA or Nrf2 siRNA, HK-2 cells were seeded in a 6-well plate (5 × 105 cells/well), treated with or without farrerol (20 μM) for 24 h, and then collected cells for western blotting.

### Statistical Analysis

The data are presented as the mean ± SEM and were analyzed using SPSS 19.0 (IBM). The experimental data were compared by one-way analysis of variance (ANOVA). Statistical significance was defined as *p* < 0.05.

## Results

### Effects of Farrerol on Cisplatin-Induced Chronic Kidney Damage

**FIGURE 1:**
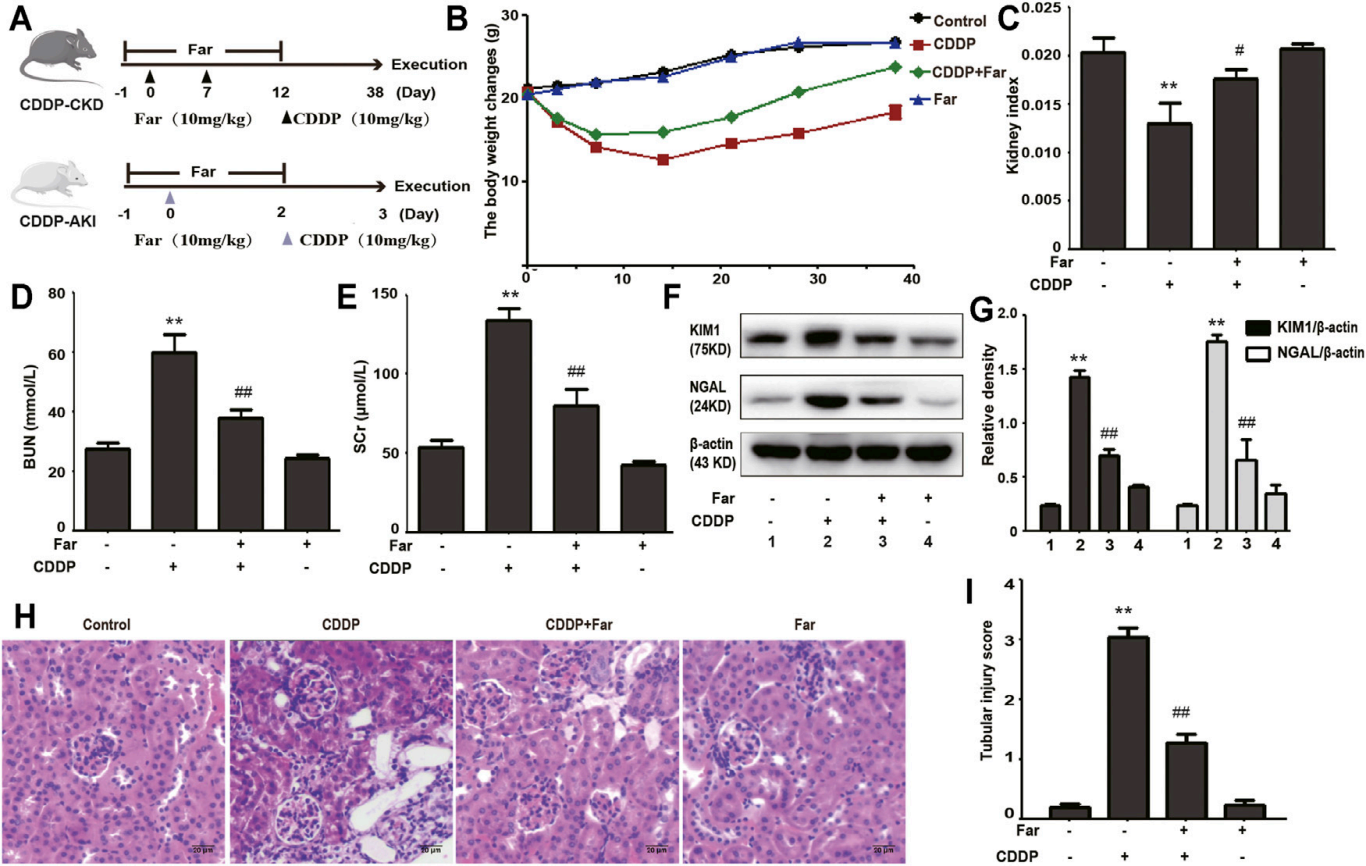
Cisplatin-induced CKD is inhibited by farrerol. **(A)** The mouse models of CDDP-induced CKD and AKI combined with farrerol were established as follows. CDDP-CKD mouse model: Mice were treated daily with farrerol (10 mg/kg/d) or vehicle beginning 1 day before the first intraperitoneal cisplatin injection until 5 days after the second cisplatin injection. Subsequently, on the 31st day following the second CDDP administration, all mice were euthanized. CDDP-AKI mouse model: On day 0, mice were given cisplatin (10 mg/kg) or vehicle, and farrerol was given daily from day -1 to day 2. Subsequently, the mice were sacrificed 1 day after the last farrerol treatment. **(B)** The body weight changes were measured by measuring the mouse weights at 0, 3, 7, 14, 21, 28, and 38 days. **(C)** The kidney index refers to the kidney weight divided by the weight on day 38. BUN **(D)** and SCr **(E)** levels were evaluated in whole blood on the 38th day of CDDP treatment. The above data are presented as the mean ± SEM (*n* = 6 in each group). **(F,G)** KIM1 and NGAL immunoblotting and densitometry normalized to *β*-actin protein were applied to assess the degree of renal tubular damage. **(H,I)** H&E-stained micrographs of kidney sections were used to quantify renal tubular damage. All experiments were performed three times. *p < 0.05 and **p < 0.01 compared with the control group; ^#^ *p* < 0.05 and ^##^ *p* < 0.01 compared with the CDDP group.

Firstly, we compared the changes in body weight, kidney index, BUN, and SCr of farrerol pretreatment before cisplatin injection and farrerol (not pretreatment) after cisplatin injection in mice to establish a reasonable mice model. As shown in Supplementary Figure S1, we found that farrerol pretreatment before cisplatin injection exert more pronounced protective effect on the kidneys than farrerol (not pretreatment) after cisplatin injection. Therefore, we chose to use farrerol pretreatment to evaluate the protective effect of farrerol against recurrent cisplatin treatment. On the day before the first intraperitoneal injection of cisplatin, the mice were pretreated with intraperitoneal farrerol (10 mg/kg) or vehicle, and farrerol administration was continued daily until 5 days after the second intraperitoneal injection of cisplatin (day -1 to day 12, 10 mg/kg). The mice were sacrificed on the 3rd and 38th days after the first injection of cisplatin to establish AKI and CKD models, respectively (Figure 1A). As shown in Figure 1B, the weight of mice treated with cisplatin decreased significantly, and farrerol significantly reversed this trend. Moreover, analysis of the kidney index also indicated that farrerol significantly improved the CKD caused by cisplatin (Figure 1C). In addition, farrerol improved CDDP-CKD, and this protective effect was demonstrated by renal index analysis (Figure 1C). In addition, farrerol pretreatment substantially alleviated the levels of BUN, SCr and the proximal tubular damage marker proteins NGAL and KIM1 (Figures 1D–G). We further elucidated the protective effect of farrerol on the mouse model by scoring H&E-stained sections and found that farrerol markedly reduced CDDP-induced histological lesions, such as tubular dilation and brush-border loss (Figures 1H,I).

### Farrerol Relieves Cisplatin-Associated Inflammation and Kidney Fibrosis *in Vivo*

**FIGURE 2:**
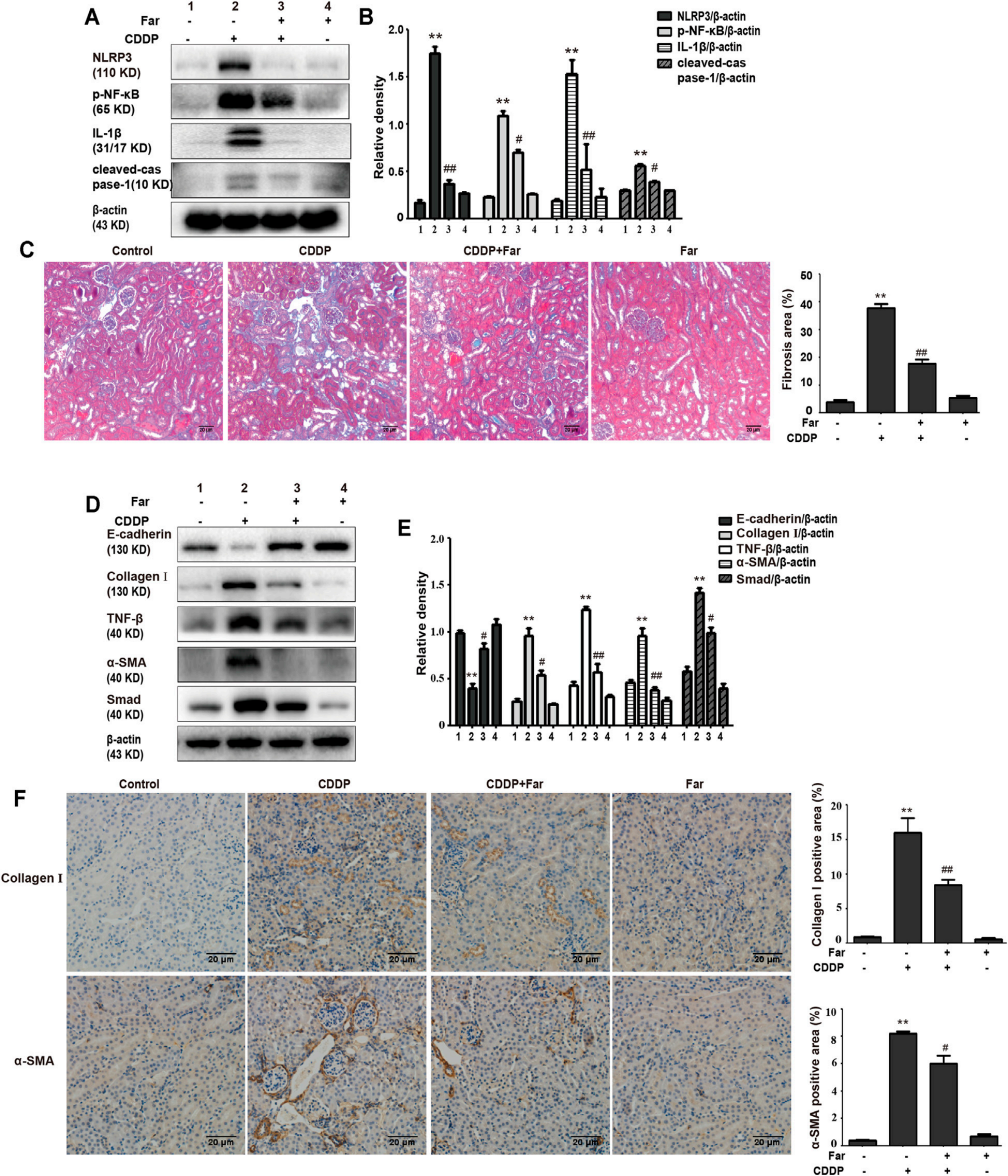
Farrerol ameliorates cisplatin-induced inflammation and kidney fibrosis in mice. The protein levels of p-NF-κB, NLRP3, cleaved caspase-1 and IL-1*β* in C57BL/6 mice were investigated by western blotting **(A)** and analyzed by densitometry analysis **(B)**. **(C)** Masson staining of renal sections and quantitative analysis of fibrosis area in the kidney tissue. Western blotting images of TGF-*β*, E-cadherin and the fibrosis-related proteins Smad, collagen I and *α*-SMA in the kidney are shown **(D)** and evaluated **(E)**. **(F)** Immunohistochemical analysis of collagen I and *α*-SMA (control *n* = 6, farrerol *n* = 6, cisplatin *n* = 6, and CDDP + farrerol = 6). A quantitative analysis of collagen I and *α*-SMA positive area in the kidney tissue. Data are shown as mean ± SEM. All experiments were performed three times. *p < 0.05 and **p < 0.01 vs. the control group; ^#^ *p* < 0.05 and ^##^ *p* < 0.01 vs. the CDDP group. *β*-actin was used as an internal control.

Notably, damaged tubular epithelial cells can cause the release of a variety of proinflammatory cytokines to induce kidney inflammation (Lorenz et al., 2014). To determine whether cisplatin induces inflammation in renal tubular epithelial cells, immunoblotting was performed to detect the levels of inflammation-mediated proteins. As shown in Figures 2A,B, cisplatin stimulation significantly increased the expression of p-NF-κB and NLRP3 and upregulated downstream cleaved caspase-1 and IL-1*β*. Furthermore, farrerol significantly reduced the inflammatory response by inhibiting p-NF-κB and its downstream targets. In addition, persistent kidney damage and unresolved inflammation may lead to failure of tissue repair, thereby promoting the development of fibrosis (Qin et al., 2016). Mice treated with cisplatin showed a significant increase in fibrosis area that was reduced with farrerol pretreatment (Figure 2C). Moreover, cisplatin-induced fibrosis led to a substantial increase in TGF-*β*, stimulated the expression of fibrosis-related proteins (such as Smad, collagen I and *α*-SMA) and decreased the level of the antifibrotic protein E-cadherin (Figures 2D,E). To further illustrate that farrerol has a therapeutic effect on cisplatin-induced fibrosis, we conducted immunostaining of collagen I and *α*-SMA and confirmed that farrerol improves renal fibrosis (Figure 2F).

### Farrerol Alleviates Cisplatin-Induced Oxidative Stress *in Vivo*

**FIGURE 3:**
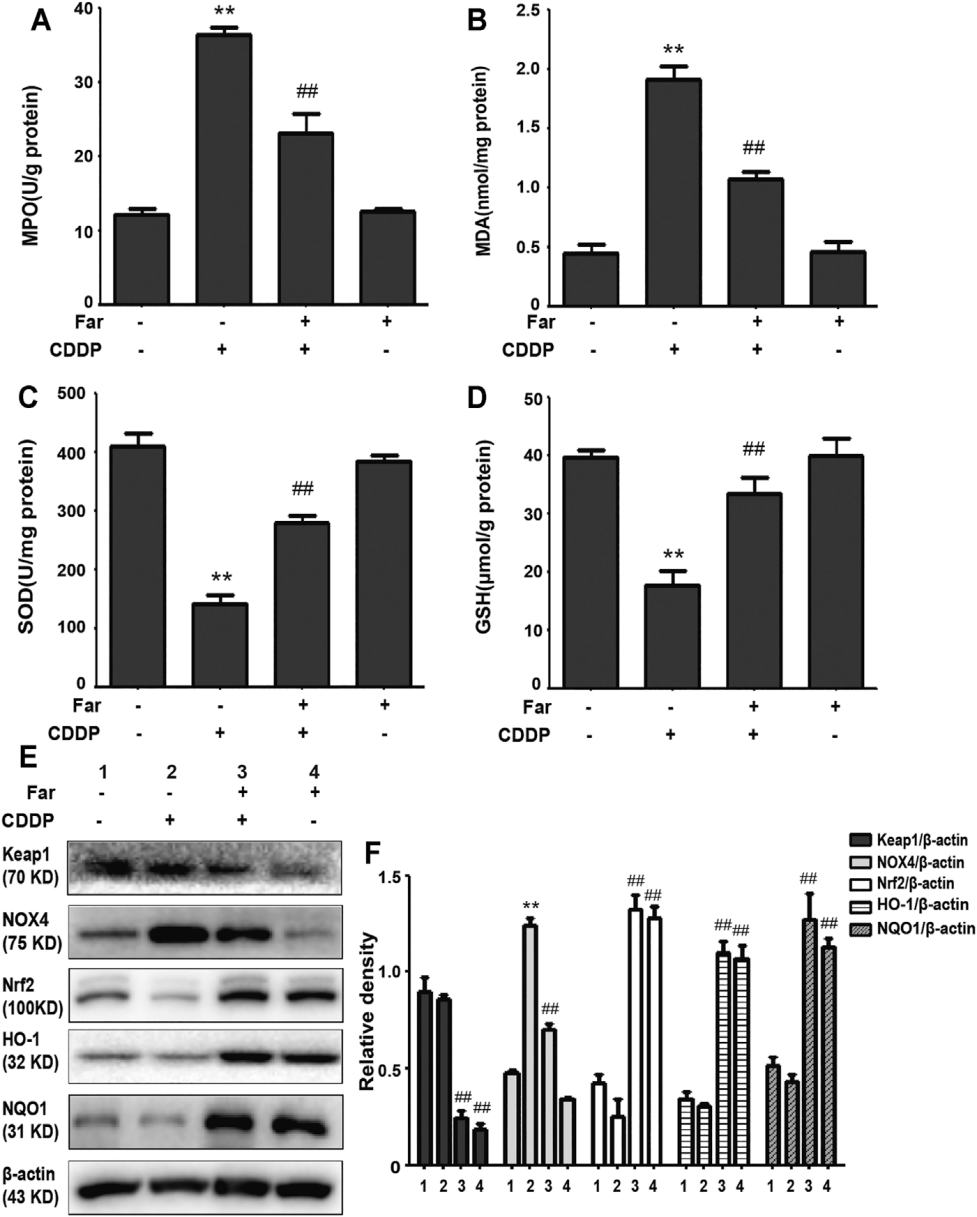
Farrerol alleviates cisplatin-induced oxidative stress *in vivo*. **(A–D)** Mice were sacrificed on the 38th day after the first cisplatin administration, and renal tissues were collected. The contents of MPO, MDA, SOD, and GSH in kidney tissue were measured. Data are expressed as the mean ± SEM (*n* = 6 per group). **(E,F)** Kidney tissue lysates were analyzed by immunoblotting with specific antibodies against Keap1, NOX4, Nrf2, HO-1, and NQO1. The expression levels of the abovementioned oxidation pathway-related proteins were quantified by densitometry and standardized to *β*-actin. Data are expressed as the mean ± SEM. *p < 0.05 and **p < 0.01 compared with the control group; ^#^ *p* < 0.05 and ^##^ *p* < 0.01 compared with the CDDP group.

Repeated stimulation with cisplatin induces the production and accumulation of excess ROS in the proximal tubules of the kidney, causing an imbalance in the body’s redox system. After pretreatment with farrerol, the contents of MDA and MPO, which are key to the ROS-induced imbalance of the CKD redox system, were greatly reduced, and the contents of the antioxidant enzymes GSH and SOD were increased (Figures 3A–D). Previous experiments have shown that the antioxidant capacity of farrerol involves the activation of Nrf2. Therefore, we examined whether the antioxidant effect of farrerol on CDDP-CKD is related to the upregulation of Nrf2-mediated signaling pathways. The results showed that farrerol can effectively activate Nrf2 and its downstream target proteins HO-1 and NQO1 while reducing the levels of Keap1 and NOX4 (Figures 3E,F).

### Farrerol Activates PINK1/Parkin-Mediated Mitophagy and Protects Against the Exacerbation of Kidney Damage in Cisplatin-induced CKD Mice

**FIGURE 4:**
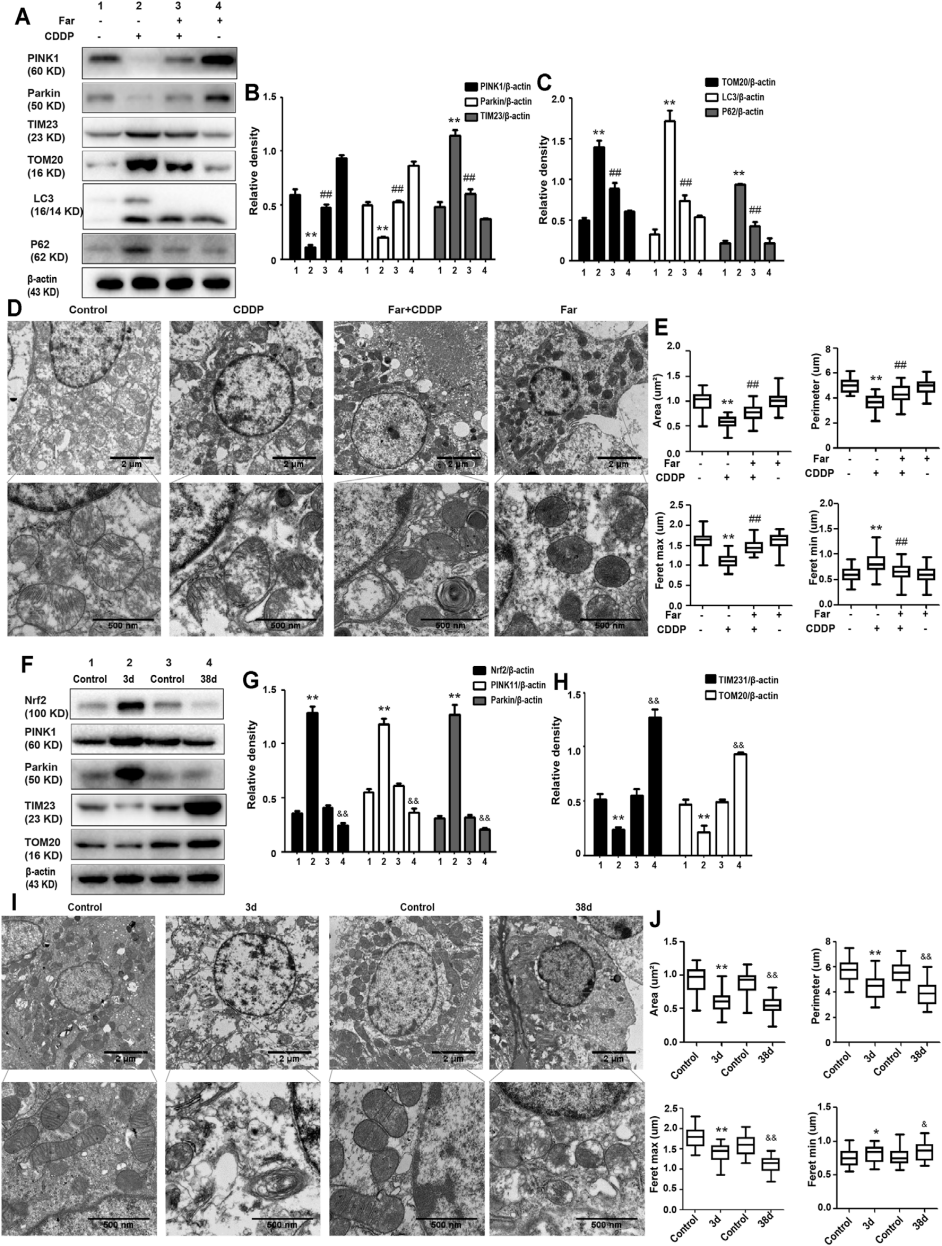
The effect of CDDP on the Nrf2- and PINK1/Parkin-related mitophagy pathways in AKI and CKD. **(A–C)** Immunoblot analysis of PINK1, Parkin, TIM23, TOM20, LC3, and P62. **(D,E)** Representative TEM images of mitochondrial morphology in renal tubular epithelial cells. Data were shown as quantification of mitochondrial contour measurements from *n* = 6 mice per group. **(F–H)** Western blotting and densitometry analysis of Nrf2, PINK1, Parkin, TIM23, and TOM20. **(I,J)** Representative TEM images of autophagosomes/mitochondria in kidney tubular epithelial cells on day 3 and day 38 after the first injection of cisplatin. All experiments were performed three times. Data were shown as quantification of mitochondrial contour measurements from *n* = 6 mice per group. Data are expressed as the mean ± SEM. ^&^ *p* < 0.05 and ^&&^ *p* < 0.01 *p < 0.05 and **p < 0.01 vs. the control group (CDDP-AKI mice); *p < 0.05 and **p < 0.01 vs. the control group (CDDP-CKD mice); ^#^ *p* < 0.05 and ^##^ *p* < 0.01 vs. the CDDP group.

Mitophagy eliminates damaged mitochondria in renal tubular cells during the process of kidney damage and repair (Lin Q. et al., 2019). Moreover, Nrf2 also restores the mitochondrial dynamics of renal tubular cells by regulating PINK1-mediated mitophagy (Xiao et al., 2017). Thus, the possible involvement of the Nrf2/PINK1-mediated mitophagy pathway was tested in our experimental model. Similar to previous results, farrerol, as a Nrf2 activator, triggered PINK1/Parkin-mediated mitophagy on the 38th day of cisplatin administration, increased the accumulation of LC3 and decreased the protein expression of translocase of mitochondrial inner membrane 23 (TIM23), translocase homolog of mitochondrial outer membrane 20 (TOM20) and P62 (Figures 4A–C). Then, we also evaluated the effect of cisplatin on mitochondria through TEM and found that farrerol can reduce mitochondrial damage and activated mitophagy in the kidney (Figures 4D,E; Table 1). To better understand the role of mitophagy in cisplatin-induced kidney injury, we compared acute and chronic cisplatin-related mouse models. In our study, we found that Nrf2, PINK1, and Parkin were significantly increased on the third day of cisplatin stimulation. Moreover, we also found accumulation of LC3II, which suggested that autophagy was activated, and reduced levels of TIM23 and TOM20, indicating mitochondrial clearance by mitophagy. Most importantly, we also found that the changes in these proteins were significantly reversed on day 38 of cisplatin stimulation (Figures 4F–H). In addition, we also observed clear autophagosomes/mitophagosomes on the 3rd day of cisplatin stimulation, but on the 38th day, we observed a large number of damaged mitochondria and almost no autophagosomes/mitophagosomes unobserved on the 38th day, accompanied by a large number of damaged mitochondria (Figures 4I,J; Table 2).

**TABLE 1:** Contour measurements of mitochondria.

Parameters	Control		Far	
	Control	CDDP	Control	CDDP
Roundness	0.467 ± 0.051	0.591 ± 0.073	0.514 ± 0.082	0.478 ± 0.065
Aspect ratio	2.503 ± 0.069	1.649 ± 0.089	2.316 ± 0.029	2.606 ± 0.059
Shape factor	5.209 ± 0.313	4.202 ± 0.305	4.91 ± 0.346	5.122 ± 0.262

**TABLE 2:** Contour measurements of mitochondria.

Parameters	3d		38d	
	Control	CDDP	Control	CDDP
Roundness	0.347 ± 0.075	0.411 ± 0.208	0.337 ± 0.069	0.526 ± 0.344
Aspect ratio	2.468 ± 0.063	1.659 ± 0.064	2.066 ± 0.062	1.361 ± 1.121
Shape factor	5.837 ± 0.746	5.235 ± 1.886	5.611 ± 0.733	4.743 ± 2.256

### Nrf2 Knockout Aggravates Cisplatin-Induced Kidney Damage and Renal Fibrosis

**FIGURE 5:**
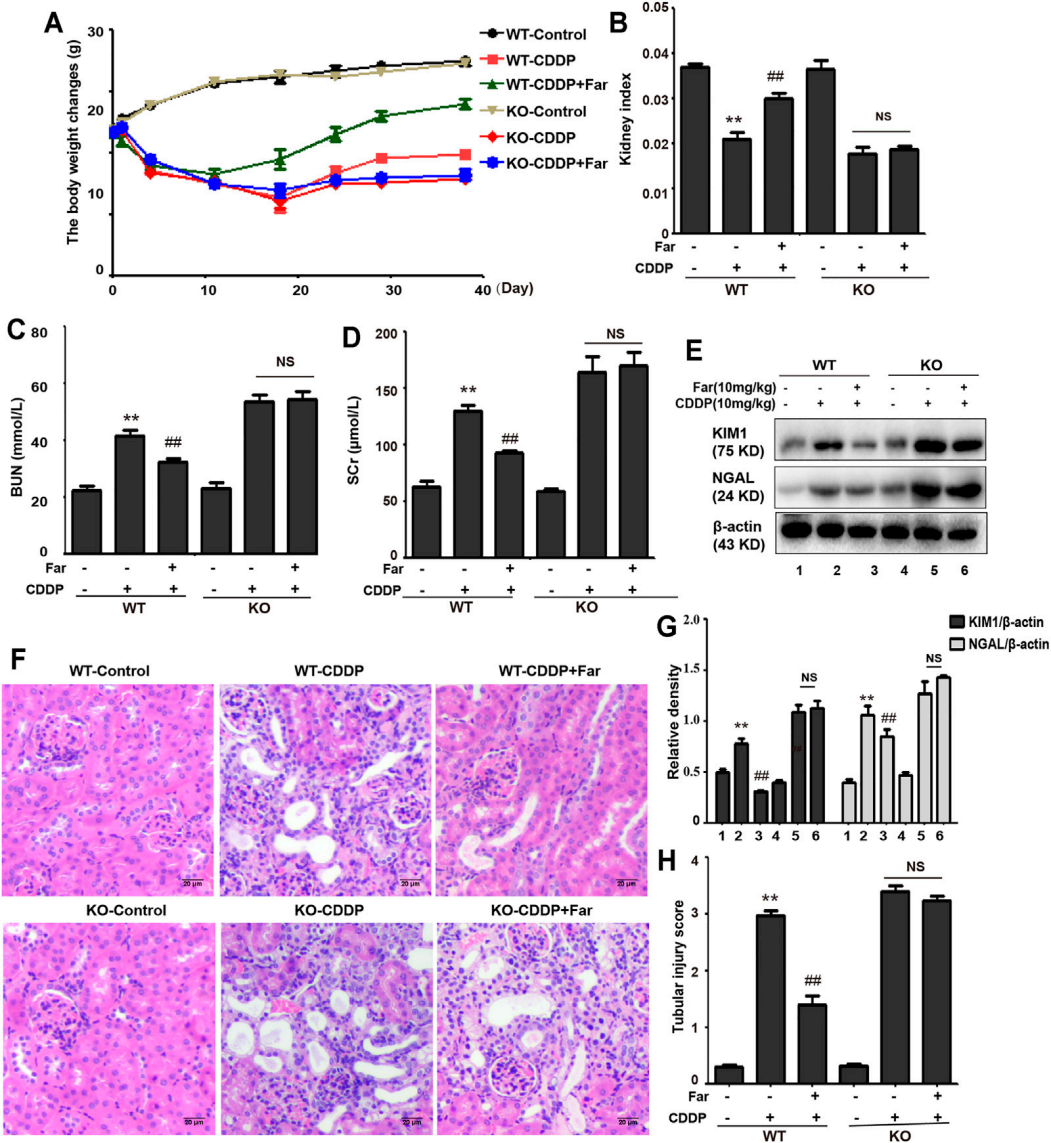
Nrf2 knockout exacerbates CDDP-induced kidney damage. C57BL/6 wild-type mice and Nrf2 knockout C57BL/6 mice were treated with cisplatin intraperitoneally on day 0 and day 7 (10 mg/kg per dose). Then, the mice were sacrificed on the 31st day after the second administration of CDDP, and kidneys and whole blood were collected. Changes in weight **(A)** and kidney index **(B)** of wild-type and Nrf2 knockout mice were evaluated. The collected whole blood was used to measure BUN **(C)** and SCr **(D)**. **(E,F)** Kidney tissue lysates were analyzed by immunoblotting with specific antibodies against KIM1 and NGAL. **(G,H)** Typical photomicrographs of kidney sections were used to quantify kidney damage. The data are shown as the mean ± SEM (*n* = 6 in each group). All experiments were performed three times. *p < 0.05 and **p < 0.01 compared with the control group; ^#^ *p* < 0.05 and ^##^ *p* < 0.01 compared with the CDDP group. NS, no specificity; WT, wild-type C57BL/6 mice; KO, Nrf2 knockout C57BL/6 mice.

**FIGURE 6:**
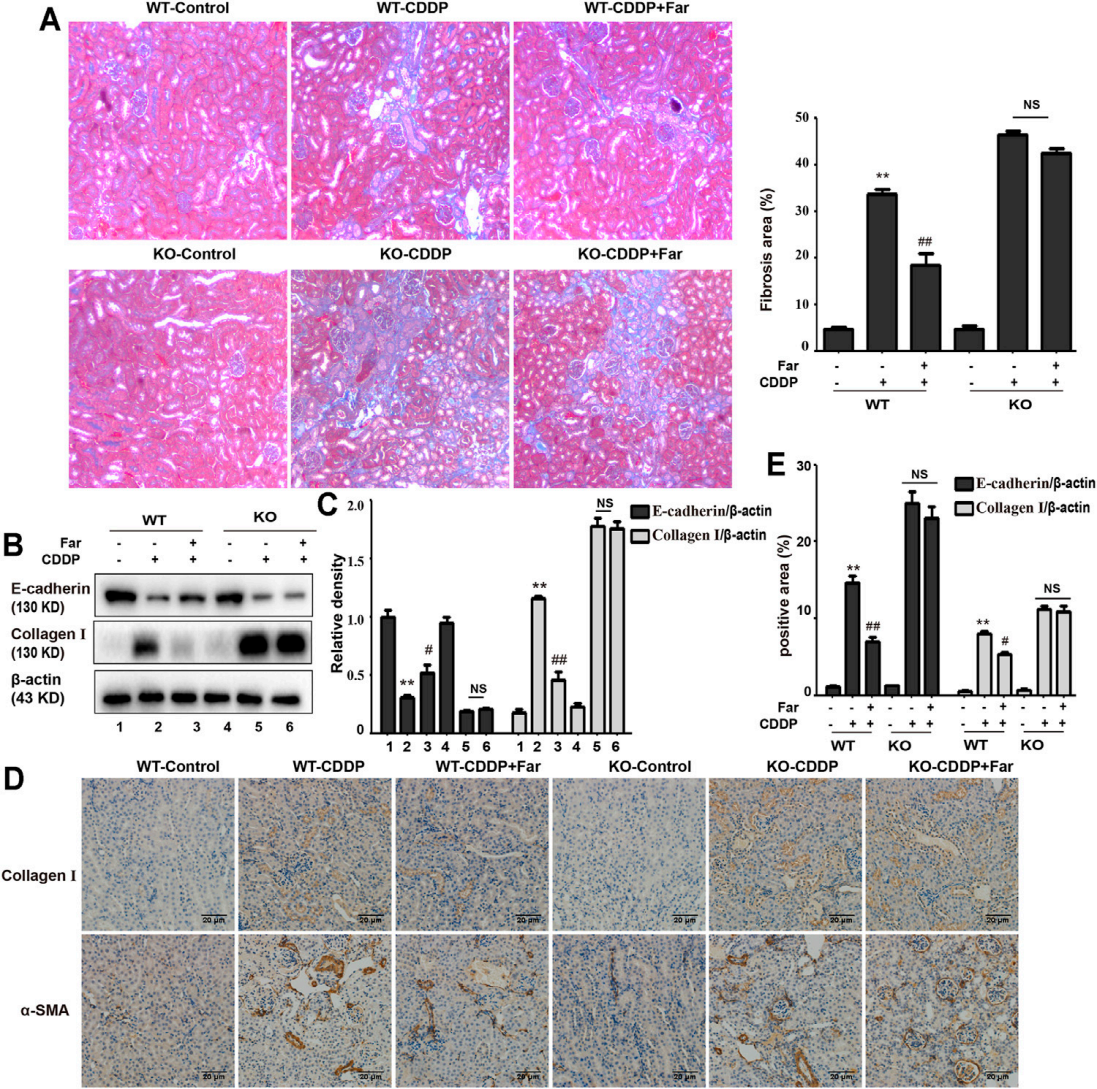
Nrf2 knockout exacerbates CDDP-induced renal fibrosis. **(A)** Masson staining was utilized to show the fibrotic areas in the kidneys of different CDDP-CKD mice. A quantitative analysis of fibrosis area in the kidney tissue. **(B,C)** Western blotting images and quantification of E-cadherin and collagen I in mouse kidney tissue lysates normalized to *β*-actin. **(D)** Representative images of collagen I and *α*-SMA immunohistochemical analysis of mouse kidneys. **(E)** A quantitative analysis of collagen I and *α*-SMA positive area in the kidney tissue. Data are shown as mean ± SEM. All experiments were conducted three times. *p < 0.05 and **p < 0.01 compared with the control group; ^#^ *p* < 0.05 and ^##^ *p* < 0.01 compared with the CDDP group. NS, no specificity; WT, wild-type C57BL/6 mice; KO, Nrf2 knockout C57BL/6 mice.

Next, we assessed whether Nrf2 knockdown exacerbates kidney damage in mice. Changes in body weight, renal function index (BUN, SCr, KIM1, NGAL) and histological characteristics (H&E staining) were measured to assess kidney function in the mouse model. Compared with that of wild-type mice, the kidney function of Nrf2 knockout mice deteriorated significantly after treatment with cisplatin (Figures 5A–H). Most importantly, these results indicated that farrerol had little protective effect on Nrf2-deficient mice. In addition, compared with the wild-type mice, the Nrf2 knockout mice pretreated with farrerol did not exhibit a decrease in the area of fibrosis. In contrast, more fibrotic areas, a decrease in the antifibrotic protein E-cadherin, and an increase in the fibrosis-related protein collagen I were observed (Figures 6A–E).

### Nrf2 Deficiency Exacerbates Oxidative Stress in Cisplatin-induced CKD Mice

**FIGURE 7:**
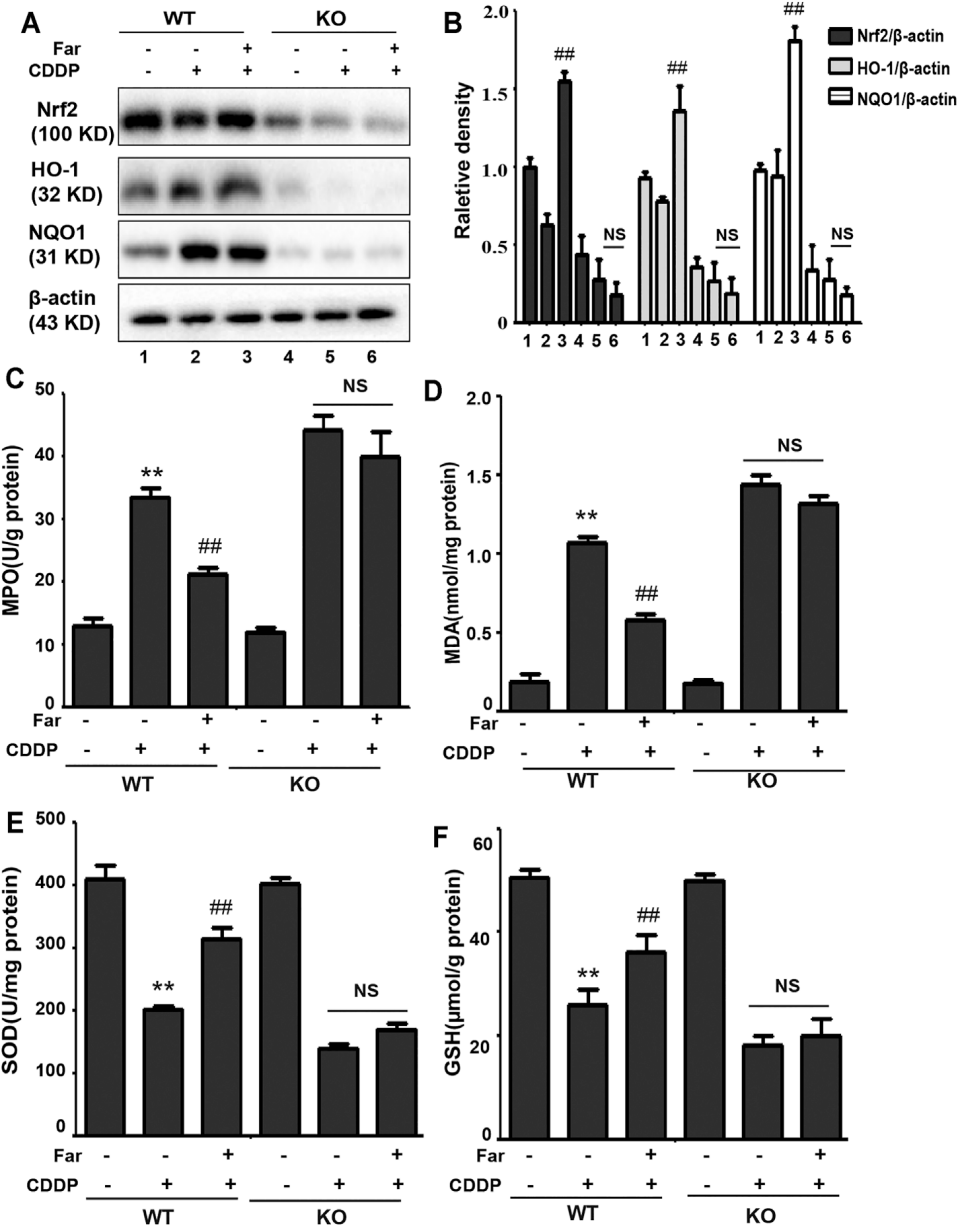
Nrf2 deficiency exacerbates oxidative stress in CDDP-CKD mouse models. **(A,B)** Western blot analysis of Nrf2, HO-1, and NQO1 in wild-type and Nrf2 knockout mouse kidneys. **(C–F)** The indicators MPO, MDA, SOD and GSH were assessed in kidney tissues. Data are expressed as the mean ± SEM (*n* = 6 per group). All of the data displayed represent the mean of three independent experiments. *p < 0.05 and **p < 0.01 vs. the control group; ^#^ *p* < 0.05 and ^##^ *p* < 0.01 vs. the CDDP group. NS, no specificity. *β*-actin was used as an internal control.

To test whether the effect of farrerol on chronic oxidative stress induced by repeated CDDP stimulation is Nrf2 dependent, we measured and compared various oxidative stress markers in Nrf2 knockout and wild-type mice. As shown in Figures 7A,B, we observed no significant changes in Nrf2 or its downstream targets HO-1 and NQO1 in wild-type mice. In addition, in Nrf2 knockout mice, the increase in MPO and MDA and the decrease in SOD and GSH content induced by cisplatin were more pronounced, and this phenomenon was not reversed by farrerol pretreatment (Figures 7C–F).

### Knockdown of Nrf2 Partially Abolishes PINK1/Parkin-Mediated Mitophagy Activated by Farrerol Pretreatment in Mice

**FIGURE 8:**
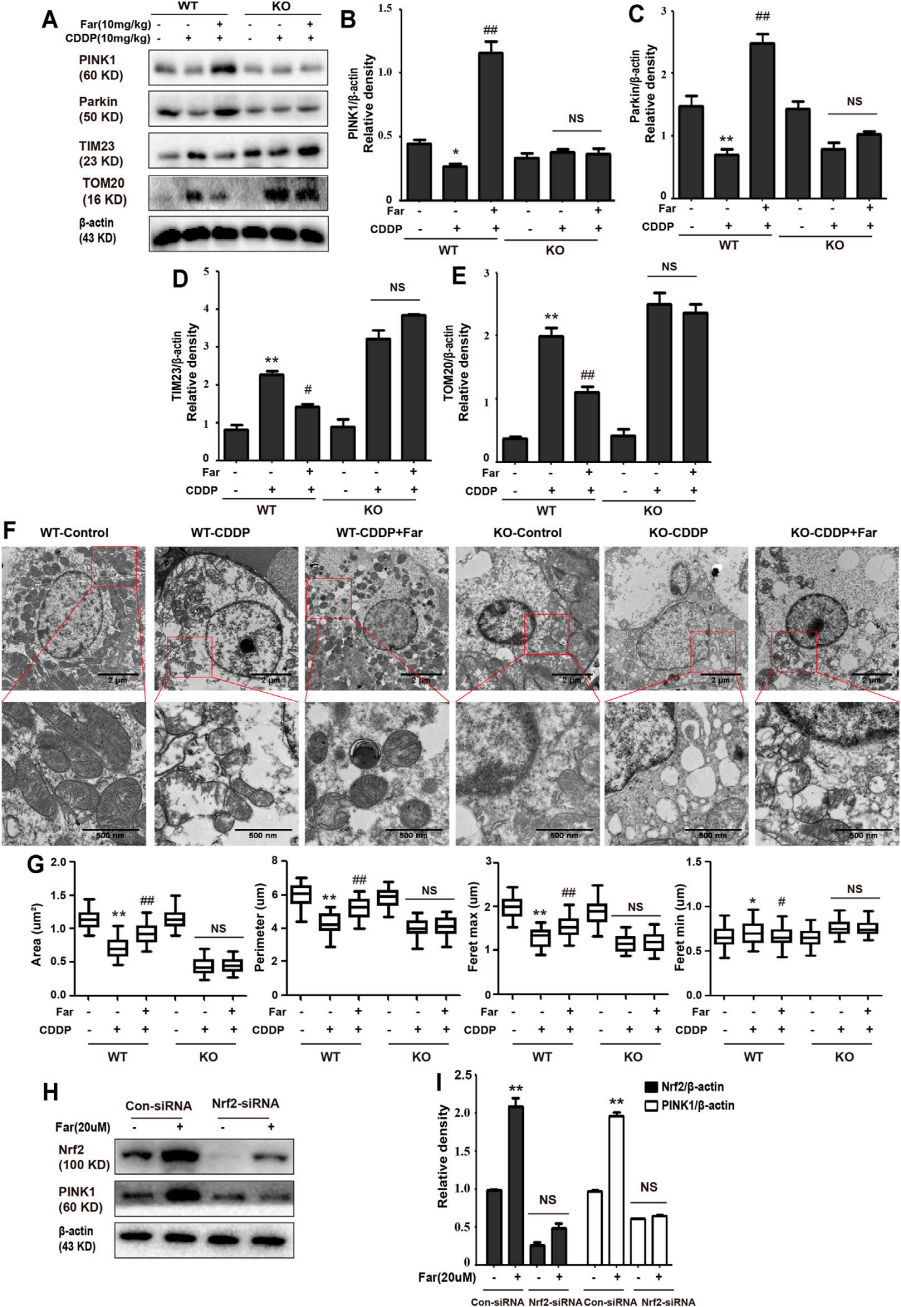
Knockdown of Nrf2 partially abolishes PINK1/Parkin-mediated mitophagy activated by farrerol pretreatment in mice. The expression levels of the mitophagy-mediating proteins PINK1, Parkin, TIM23 and TOM20 in wild-type and Nrf2 knockout mice were investigated by Western blotting **(A)** and analyzed by densitometry analysis **(B–E)**. **(F)** Representative TEM images of mitochondrial morphology in kidney tubular epithelial cells of wild-type and Nrf2 knockout mice. **(G)** Data were shown as quantification of mitochondrial contour measurements from *n* = 6 mice per group. Data are expressed as the mean ± SEM. (H, I) HK-2 cells were transfected with a Nrf2 siRNA or Nrf2-negative control siRNA for 72 h and exposed to farrerol (20 μM)for 24 h, and the protein levels were subsequently detected with western blotting. The levels of Nrf2 and PINK1 were determined. *p < 0.05 and **p < 0.01 compared with the control group; ^#^ *p* < 0.05 and ^##^ *p* < 0.01 compared with the CDDP group. NS, no specificity. *β*-actin was used as an internal control.

Nrf2 null mice and wild-type mice were utilized to further explore the relationship between Nrf2 and PINK1. After analyzing the Western blot in Figures 8A–E, we found that farrerol did not upregulate the expression of the mitophagy-related proteins PINK1 and Parkin in Nrf2 knockout mice but resulted in an increase in TIM23 and TOM20 protein expression, which indicated that the knockout of the Nrf2 gene partially eliminated PINK1/Parkin-mediated mitophagy. Furthermore, TEM analysis showed that mitochondria were more damaged in Nrf2 knockout mice, and autophagosomes/mitophagosomes were hardly observed (Figures 8F,G; Table 3). These data indicated that the protective effect of farrerol against CDDP-CKD is mediated *via* activation of Nrf2 and PINK1/Parkin-mediated mitophagy. In order to directly observe the relationship between Nrf2 expression regulation and PINK1 transcription, based on the previous experiment (Ma et al., 2019), we treated the HK-2 cells with farrerol for 24 h. The results showed that farrerol could hardly activate the protein expression of PINK1. This result further proveed the above conclusion that Nrf2 can play a role mainly by regulating the expression of PINK1(Figures 8H,I).

**TABLE 3:** Contour measurements of mitochondria.

Parameters	Nrf2-WT			Nrf2-KO		
	Control	CDDP	CDDP + Far	Control	CDDP	CDDP + Far
Roundness	0.395 ± 0.115	0.583 ± 0.242	0.501 ± 0.140	0.427 ± 0.267	0.604 ± 0.298	0.596 ± 0.337
Aspect ratio	3.11 ± 0.628	1.855 ± 0.268	2.461 ± 0.717	2.994 ± 0.713	1.568 ± 0.481	1.581 ± 0.311
Shape factor	5.588 ± 0.852	4.101 ± 1.118	4.723 ± 1.047	5.466 ± 0.609	3.952 ± 1.452	4.086 ± 1.316

## Discussion

The development of CKD is defined as a progressive decline in the glomerular filtration rate accompanied by the loss of kidney function and the accumulation of fibrous tissue. As a multifactorial disorder, the origin of CKD mainly involves diabetes, glomerulonephritis, kidney stones, drugs, and nephrotoxin (Levey and Coresh, 2012; Hill et al., 2016). Deterioration of renal function can be triggered by the nephrotoxicity of many therapeutic drugs, among which cisplatin is an important drug that causes acute and chronic kidney injury related to nephrotoxicity (Lin T. et al., 2019). Although cisplatin is a clinically effective chemotherapeutic drug, due to its nephrotoxicity, multicycle administration of cisplatin can cause permanent loss of kidney function. Even after successful cisplatin treatment, severe and life-limiting CKD may occur (Pabla and Dong, 2008). The transcription factor Nrf2, a regulator of cytoprotective proteins driven by ARE, is an essential factor in adjusting cell redox homeostasis. It has been reported that compared with wild-type mice, Nrf2-deficient mice have markedly deteriorated kidney function, as indicated by enhancements in BUN and SCr, more serious histological injury, and a higher tubular damage score (Liu et al., 2009). In our experiments, we also compared the changes in kidney function of Nrf2-null mice and wild-type mice after multiple injections of cisplatin and found an identical trend (Figures 5A–D,G,H). In addition, we tested proximal tubule damage markers and found that the levels of KIM1 and NGAL in Nrf2 knockout mice were significantly increased (Figures 5E,F). This result also supported that Nrf2 knockout mice are more sensitive to cisplatin and are more likely to suffer severe kidney damage. Farrerol, a new type of 2,3-dihydroflavonoid isolated from rhododendron, has been shown in our previous experiments to improve cisplatin-mediated AKI by upregulating Nrf2. Moreover, pretreatment with farrerol ameliorated cisplatin toxicity in Nrf2 wild-type mice but exerted little protective effect on Nrf2 knockout mice (Ma et al., 2019).

When a large amount of cisplatin accumulates in epithelial tubule cells, it can induce excessive ROS production, which causes oxidation reactions and renal damage. Malondialdehyde (MDA) and myeloperoxidase (MPO) levels are crucial to the imbalance between the accentuated pro-oxidant and deficient antioxidant capacity that occurs in CKD (Ma et al., 2019). Our experiments indicated that farrerol lowered the levels of MDA and MPO and enhanced the levels of GSH and SOD (Figures 3A–D). Additionally, damaged renal tubular epithelial cells can trigger a variety of proinflammatory factors to induce kidney inflammation. NF-κB is a heterodimer composed of p50 and p65 that can activate the NLRP3 inflammasome and mediate inflammation. In this study, farrerol significantly inhibited the release of proinflammatory mediators and protected kidney function. Our experiments further showed that this protective effect was achieved by inhibiting p-NF-κB and NLRP3 and reducing cleaved caspase-1 and IL-1*β* (Figures 2A,B). Moreover, successive kidney damage or chronic unresolved inflammation may cause tissue repair failure and promote the formation of renal fibrosis. In cisplatin-induced fibrosis, the activation of the TGF-*β* pathway released regulatory factors that further promoted the excessive expression of Smad, collagen I and *α*-SMA (Figures 2D–F). In addition, we found that farrerol effectively inhibited the fibrosis process and improve CDDP-CKD (Figure 2C). Related research has emphasized that mitochondrial pathology is crucial in AKI development and kidney repair after AKI. Therefore, timely elimination of injured mitochondria in renal tubular cells represents an important quality control mechanism for cell homeostasis and survival during kidney damage and repair (Ralto et al., 2020). Mitophagy, a selective form of autophagy, specifically eliminates excessive or damaged mitochondria. Previous studies have shown that inhibiting mitophagy induces a decline in mitochondrial function and enhances CDDP-AKI, while activating mitophagy protects cells from mitochondrial dysfunction and cisplatin-induced cell damage (Jiang et al., 2012; Zhao et al., 2017). In our mouse model, the possible involvement of the PINK1/Parkin-mediated mitophagy pathway was tested. As shown in Figures 4F–J and Table 2, immunoblotting showed that the levels of PINK1 and Parkin were significantly increased and the expression of the mitochondrial membrane proteins TIM23 and TOM20 was reduced on the third day of cisplatin stimulation. Most importantly, we also found that the levels of these proteins were significantly reduced on day 38. Similar to previous results, farrerol, a Nrf2 activator, triggered the PINK1/Parkin-mediated mitophagy pathway on the 38th day of cisplatin administration (Figures 4A–E; Table 1). Moreover, Nrf2-mediated PINK1 transcriptional regulation restores impaired mitophagy and abnormal mitochondrial dynamics in renal tubular cells (Xiao et al., 2017). Therefore, to further explore the relationship between Nrf2 and PINK1, we used Nrf2 null mice and wild-type mice and found that farrerol did not significantly activate mitophagy-related indicators in Nrf2 knockout mice (Figures 8A–G; Supplementary Figure S1; Table 3). These data indicated that the protective effect of farrerol against CDDP-CKD is mediated *via* activation of Nrf2 and PINK1/Parkin-mediated mitophagy.

## Conclusion

**FIGURE 9:**
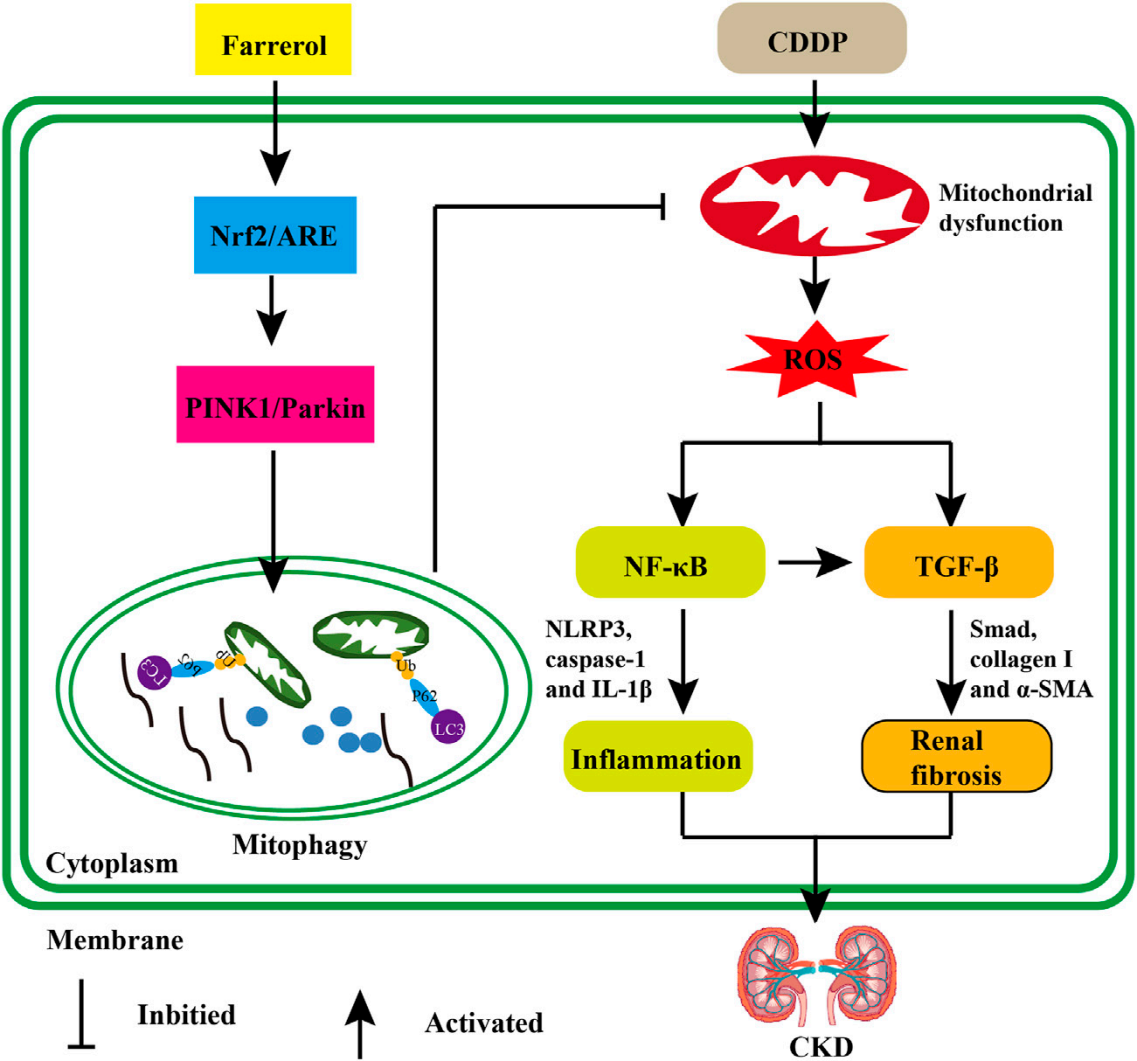
The protective effect of farrerol against CDDP-related CKD and its potential mechanism. Cisplatin can accumulate in epithelial tubule cells, causing mitochondrial dysfunction and excessive ROS. Damaged cells can induce inflammation by triggering the activation of p-NF-κB/NLRP3. In addition, the generated ROS or chronic unresolved inflammation can also stimulate the production of renal fibrosis and the development of CKD *via* TGF-*β*/Smad signaling. Farrerol can activate Nrf2/ARE, then increase PINK1/Parkin-mediated mitophagy and eliminate damaged mitochondria.

In summary, our research showed that farrerol reversed oxidative stress, inflammation and fibrosis in renal tubular epithelial cells, thereby improving cisplatin-mediated renal insufficiency. This protective mechanism of the kidney can be achieved by activating Nrf2 and subsequently increasing PINK/Parkin-mediated mitophagy and eliminating damaged mitochondria (Figure 9). These experiments demonstrated that farrerol provides a potential novel treatment for CDDP-CKD.

## Data Availability

The original contributions presented in the study are included in the article/Supplementary Material, further inquiries can be directed to the corresponding author.

## Abbreviations

*α*-SMA, alpha smooth muscle actin; AKI, acute kidney injury; ARE, antioxidant response elements; CDDP, Cisplatin; CDDP-AKI, cisplatin-induced AKI; CDDP-CKD, cisplatin-induced CKD; CKD, chronic kidney disease; GSH, glutathione; H&E, hematoxylin and eosin; HO-1, heme oxygenase-1; MDA, malondialdehyde; MPO, myeloperoxidase; NQO1, NAD(P)H quinone oxidoreductase 1; Nrf2, nuclear factor erythrocyte 2-related factor 2; ROS, reactive oxygen species; sMaf, small Maf; SOD, superoxide dismutase; TEM, tumor growth factor *β*; TGF-*β*, tumor growth factor *β*; TIM23, translocase of mitochondrial inner membrane 23; TOM20, translocase homolog of mitochondrial outer membrane 20.
