# Supplementary material for: Farrerol Ameliorated Cisplatin-Induced Chronic Kidney Disease Through Mitophagy Induction via Nrf2/PINK1 Pathway
Source: Front Pharmacol. 2021 Nov 11;12:768700. doi: 10.3389/fphar.2021.768700 (PMC8631930; doi:10.3389/fphar.2021.768700)

RAW DATA—Western blot

Fihure1

KIM1:

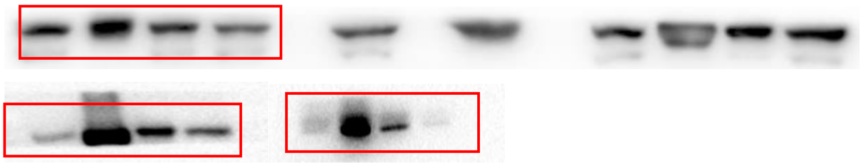

NGAL:

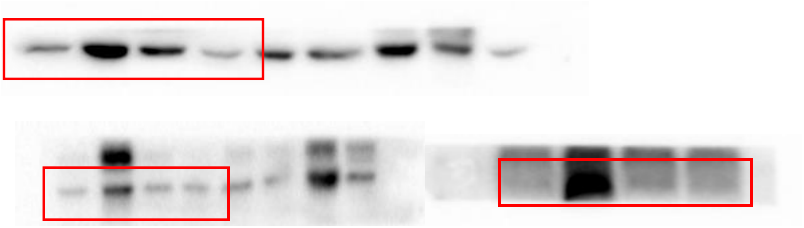

β-actin

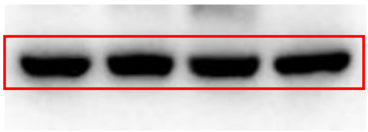

Figure2.

NLRP3

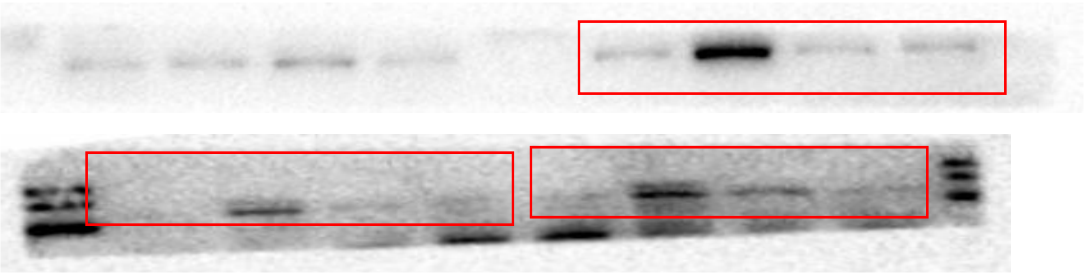

P-NFKB

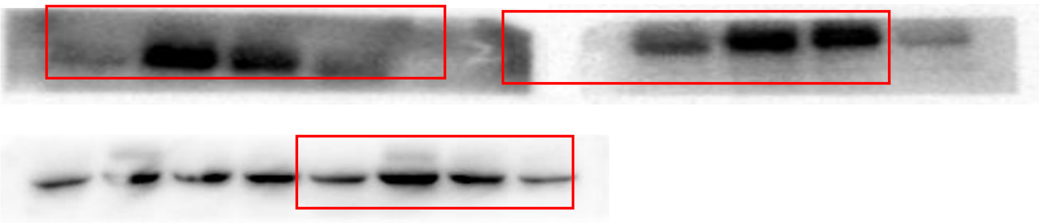

IL-1B

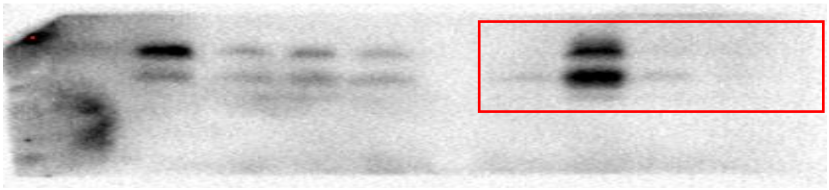

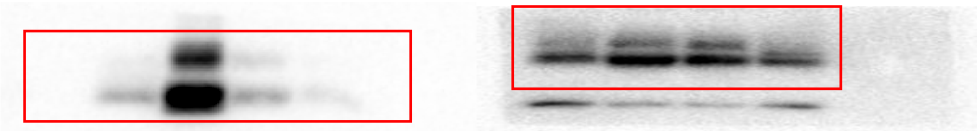

CASPASE-1

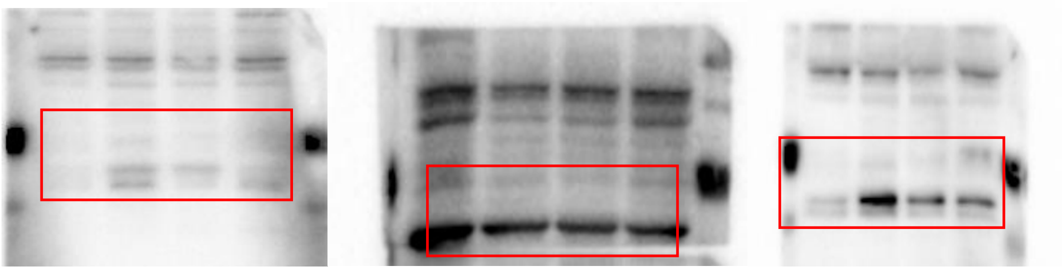

β-actin

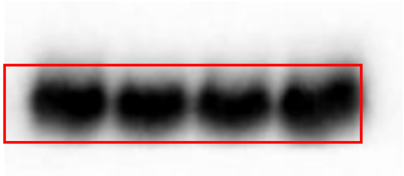

E-cad

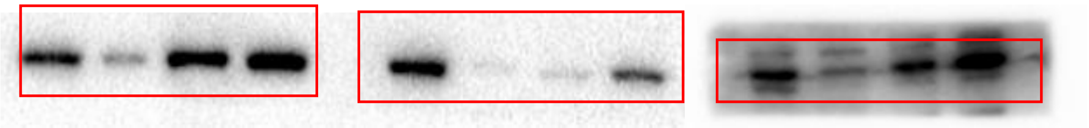

ColI

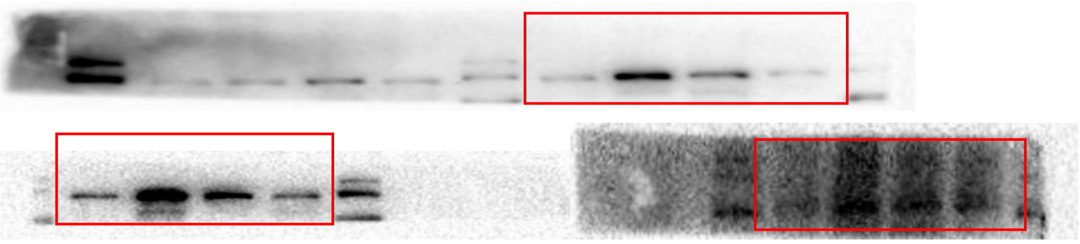

TGF-B

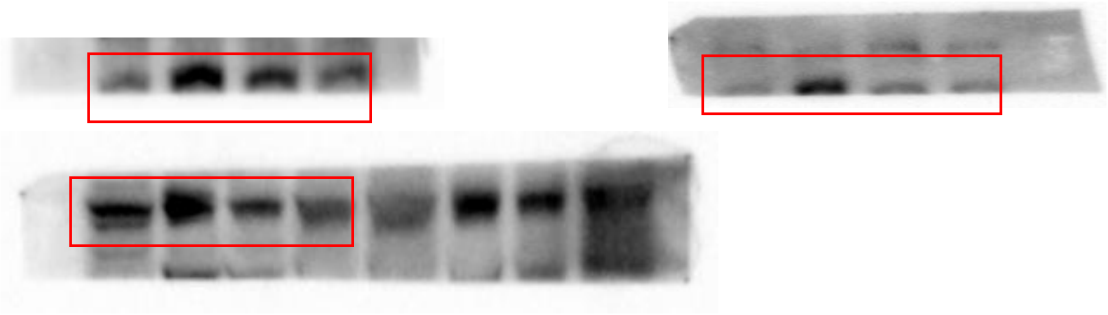

SMAD

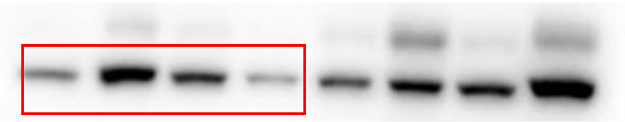

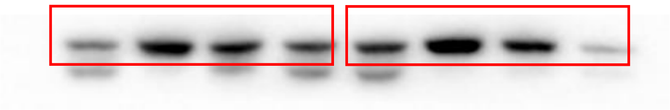

SMA

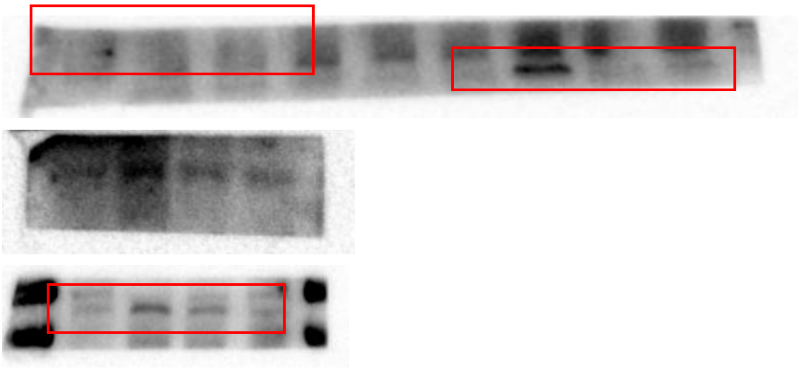

β-actin

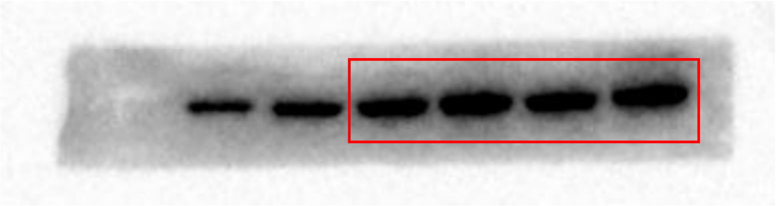

Figure3

Keap1

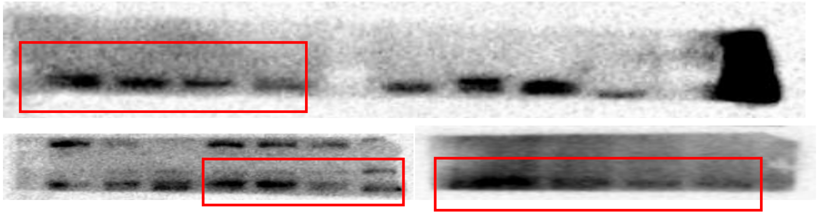

NOX4

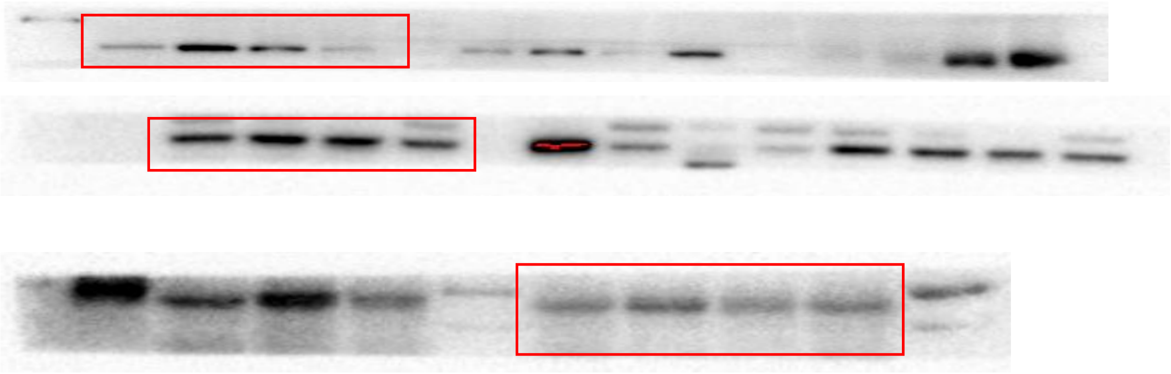

Nrf2

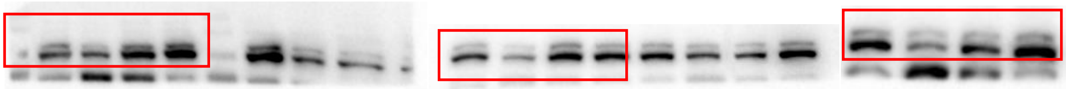

HO-1

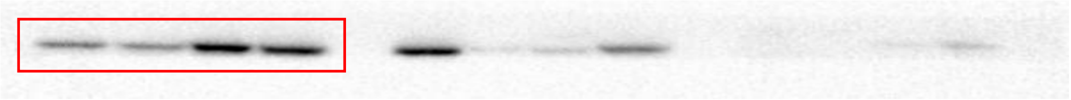

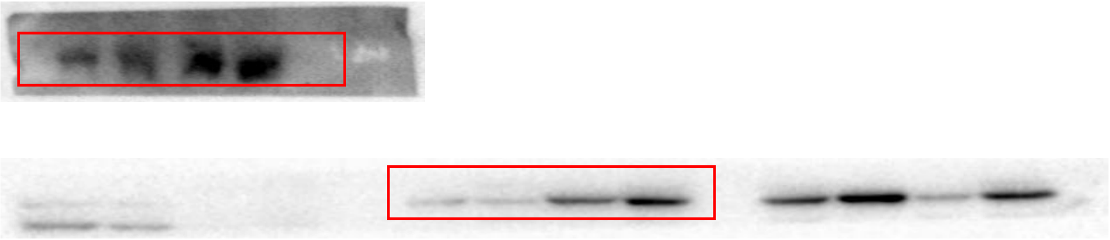

NQO1

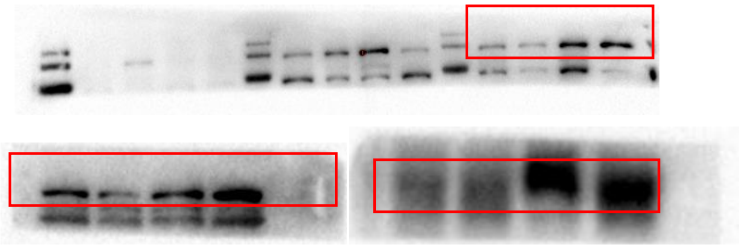

β-actin

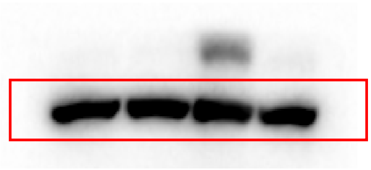

Figure4

PINK1

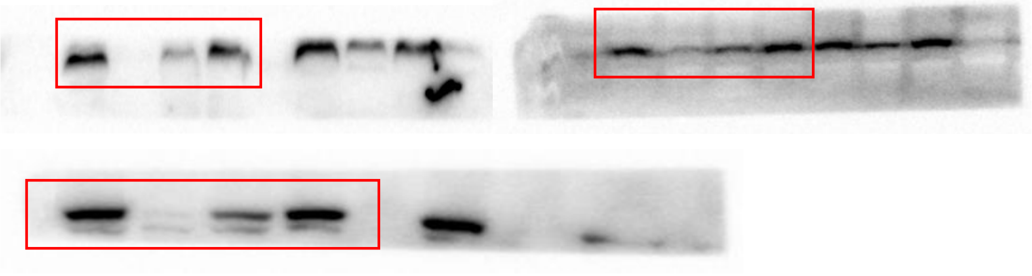

PARKIN

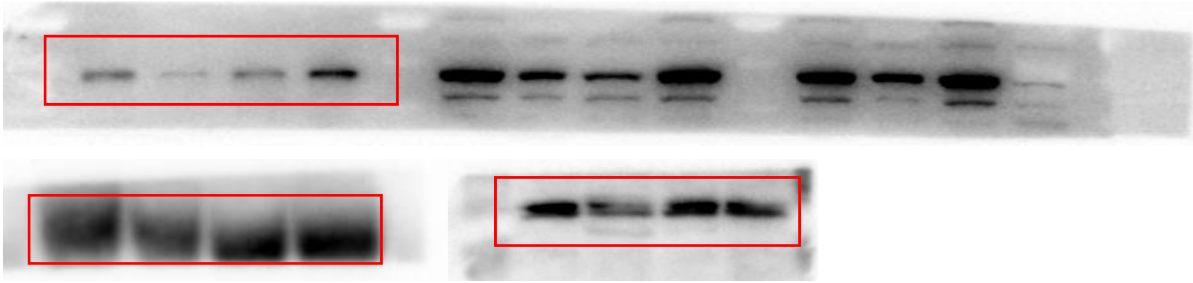

TIM23

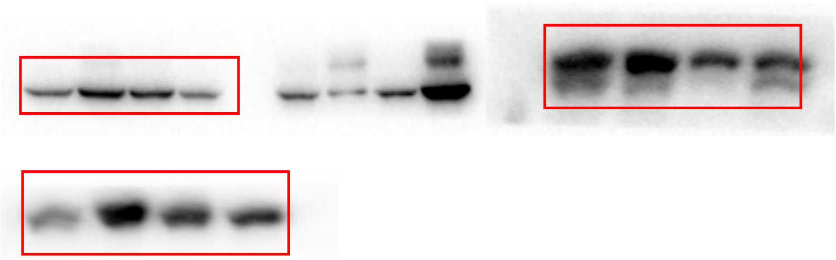

TOM20

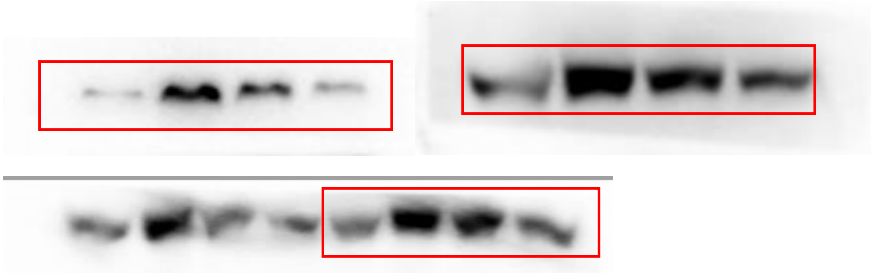

LC3

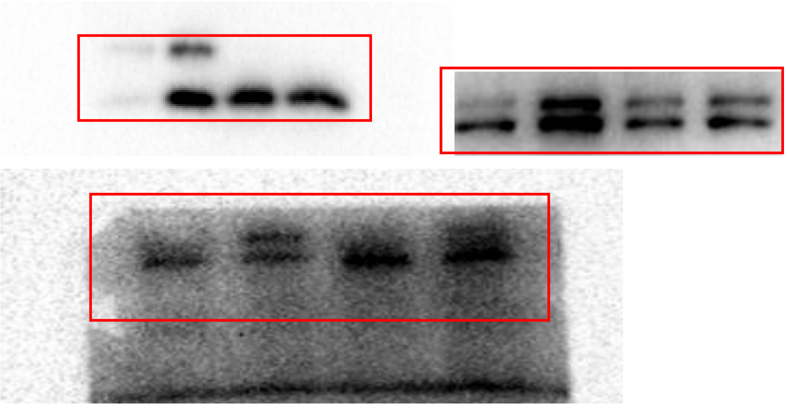

P62

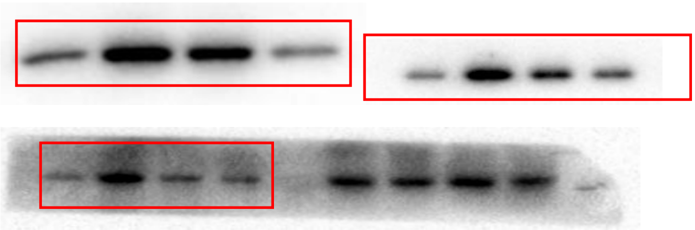

β-actin

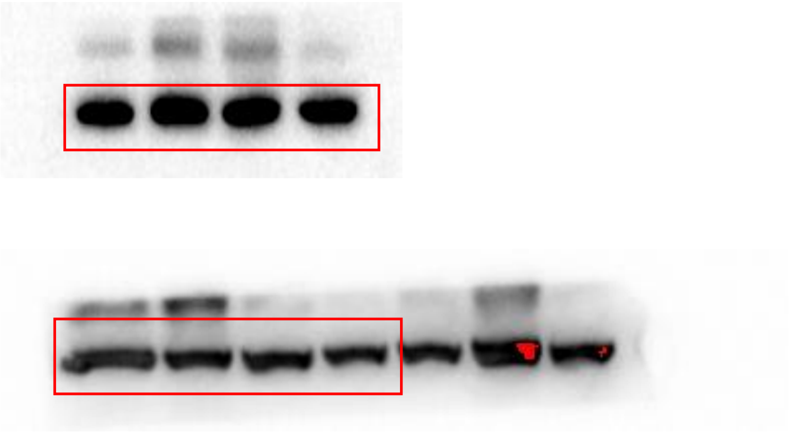

Nrf2

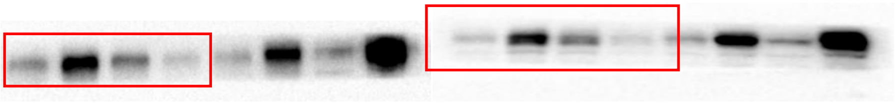

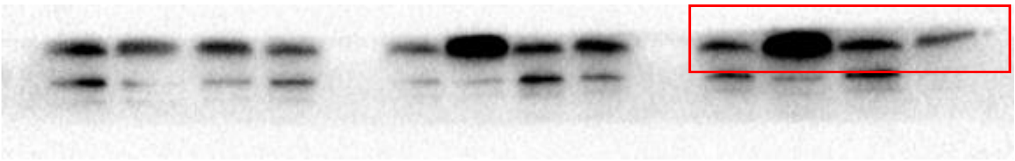

PINK

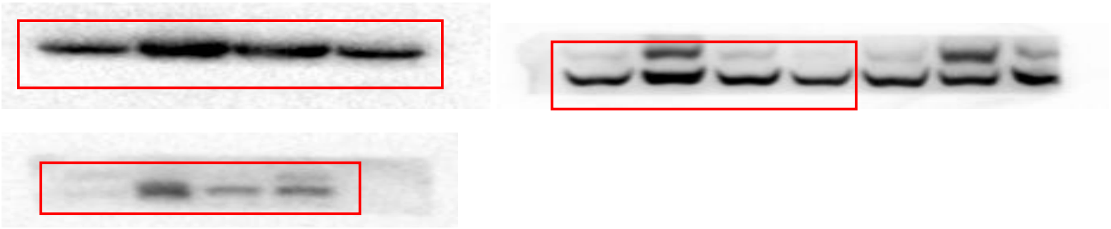

Parkin

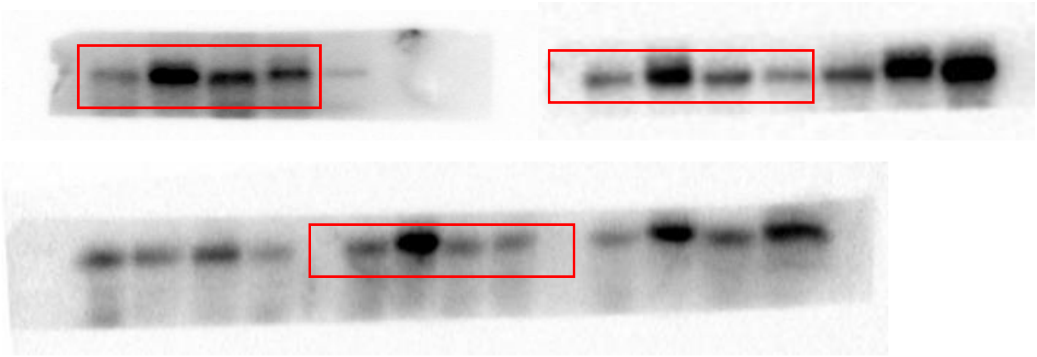

TIM23

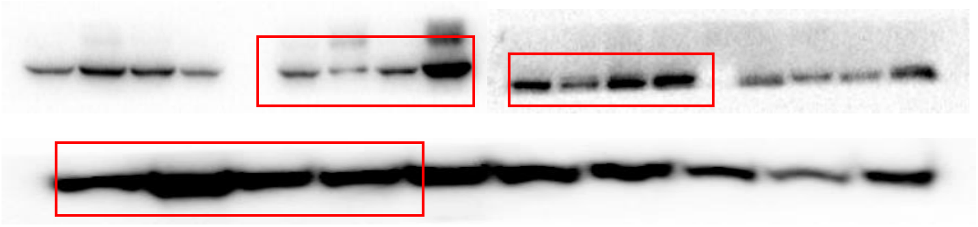

TOM20

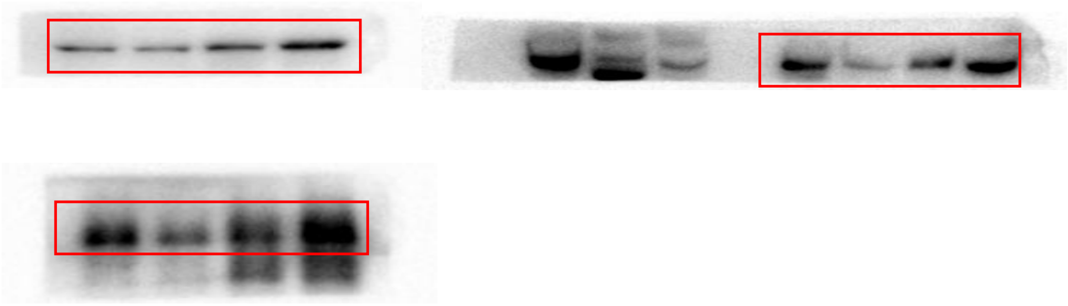

β-actin

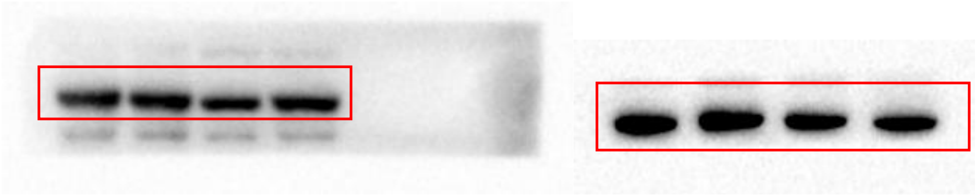

Figure5

KIM1

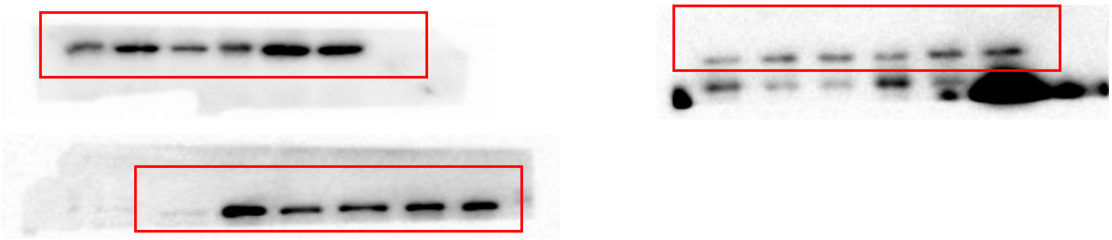

NGAL

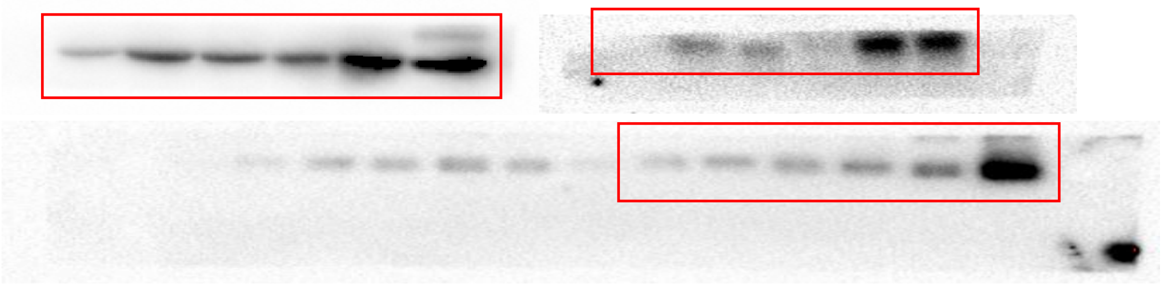

β-actin

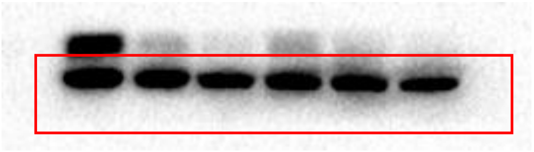

Figure6

E-cadherin

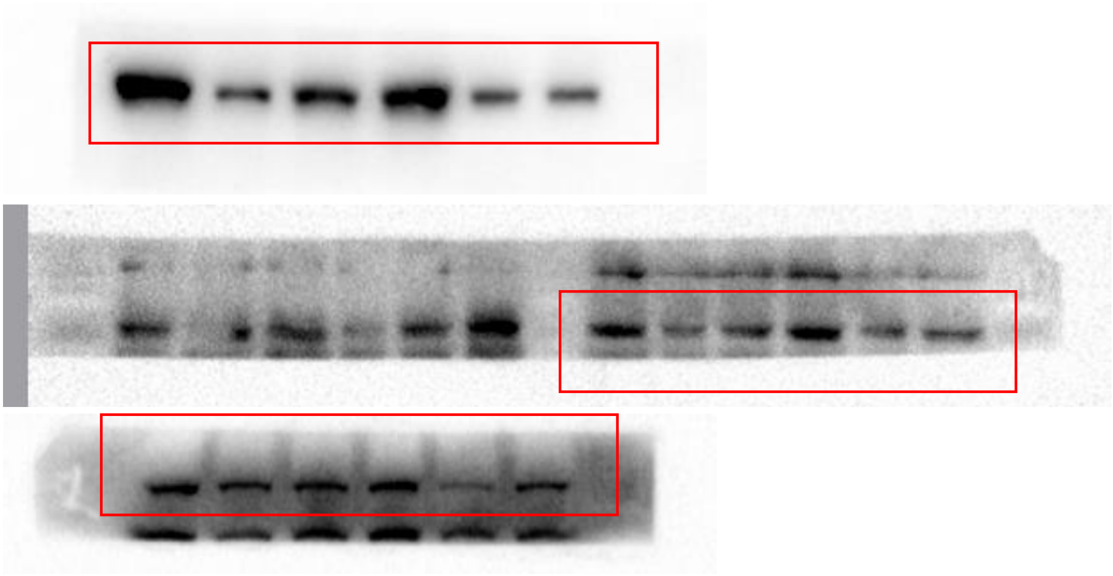

COLI

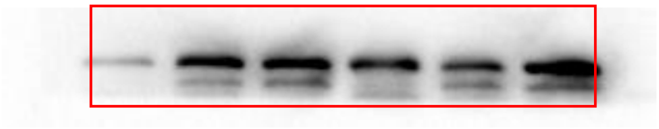

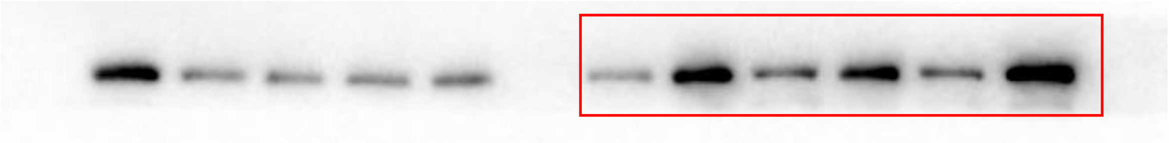

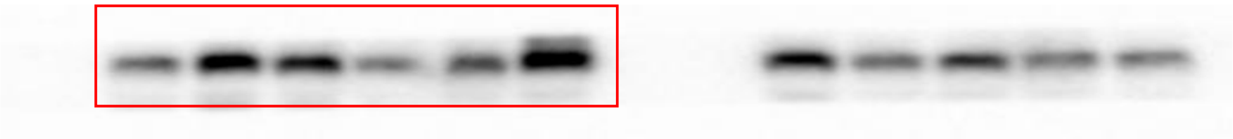

β-actin

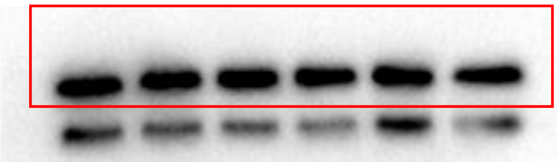

Figire7

Nrf2

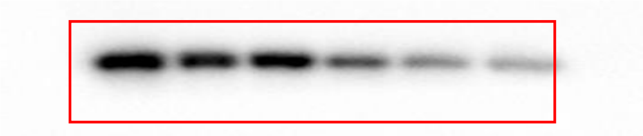

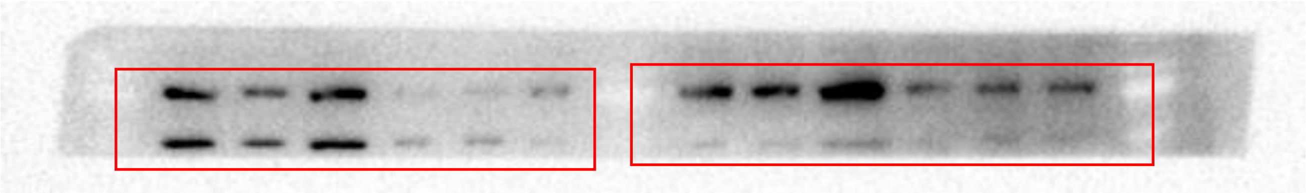

HO1

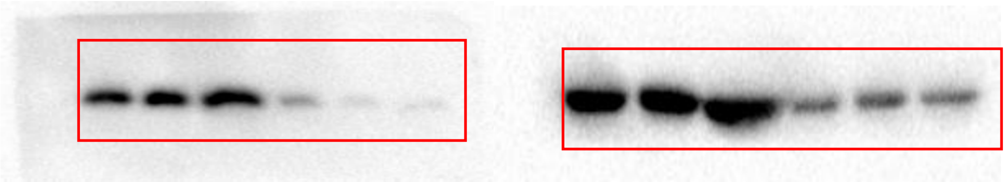

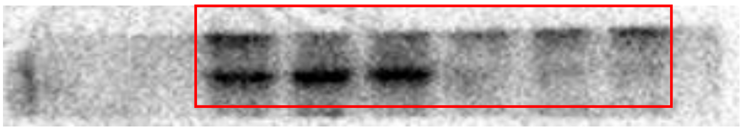

NQO1

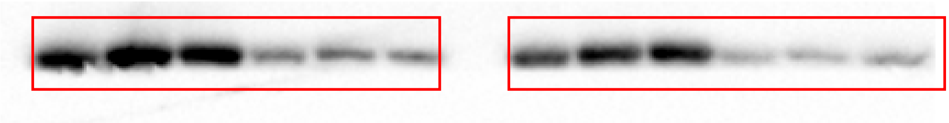

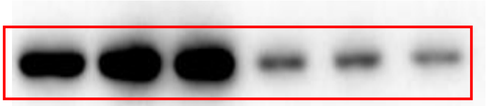

β-actin

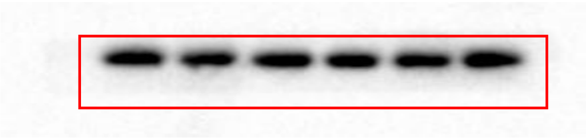

Figure8

Pink

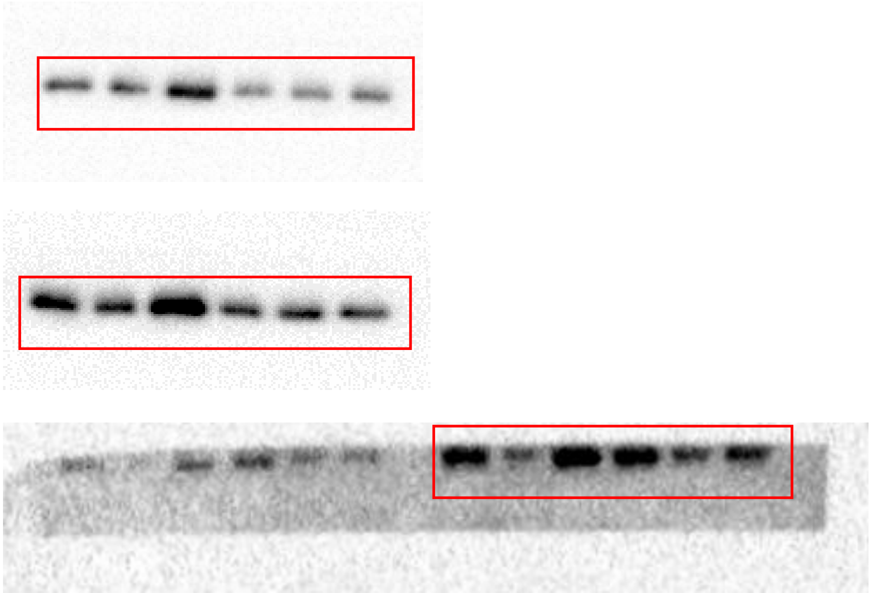

Parkin

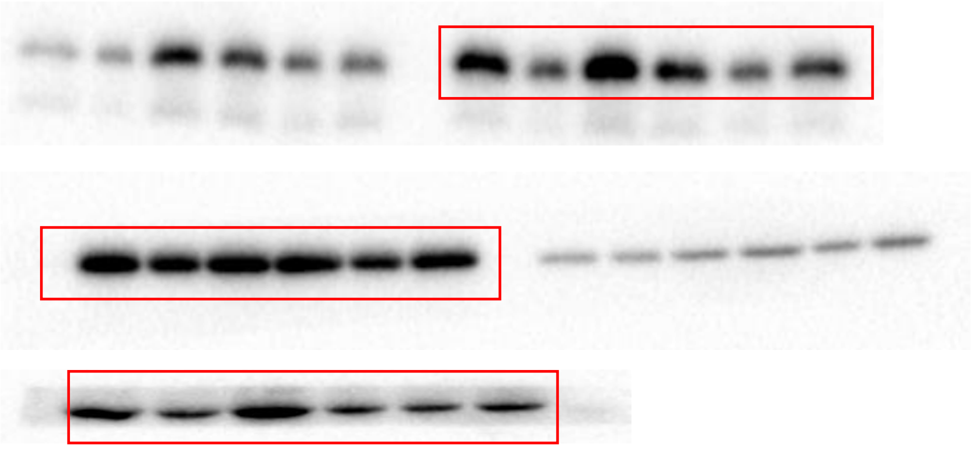

TIM23

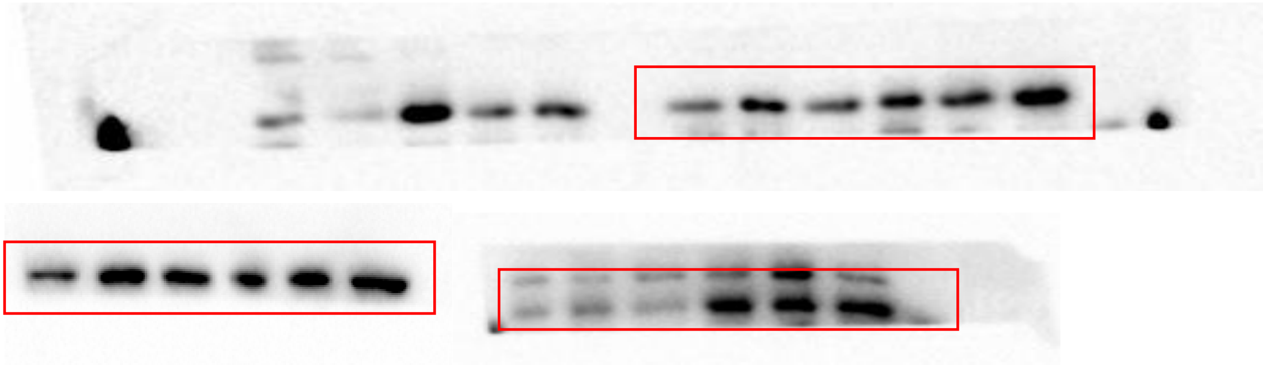

TOM20

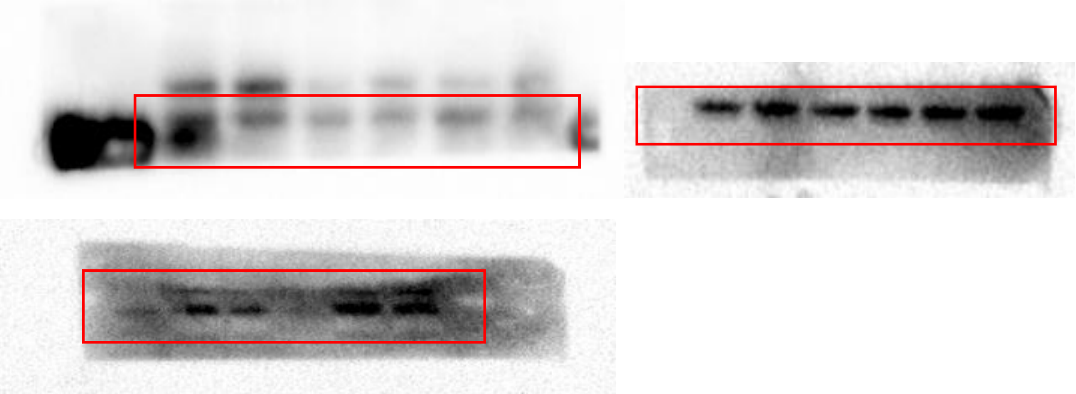

β-actin

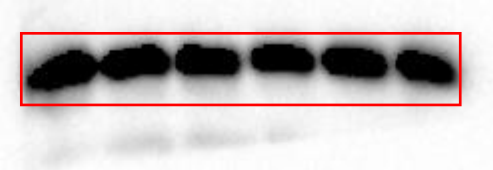

Supplement: Supplementary file 1 [file DataSheet1.PDF]
