# Supplementary material for: Farrerol Ameliorated Cisplatin-Induced Chronic Kidney Disease Through Mitophagy Induction via Nrf2/PINK1 Pathway
Source: Front Pharmacol. 2021 Nov 11;12:768700. doi: 10.3389/fphar.2021.768700 (PMC8631930; doi:10.3389/fphar.2021.768700)
Supplement: Supplementary file 2 [file Image1.pdf]

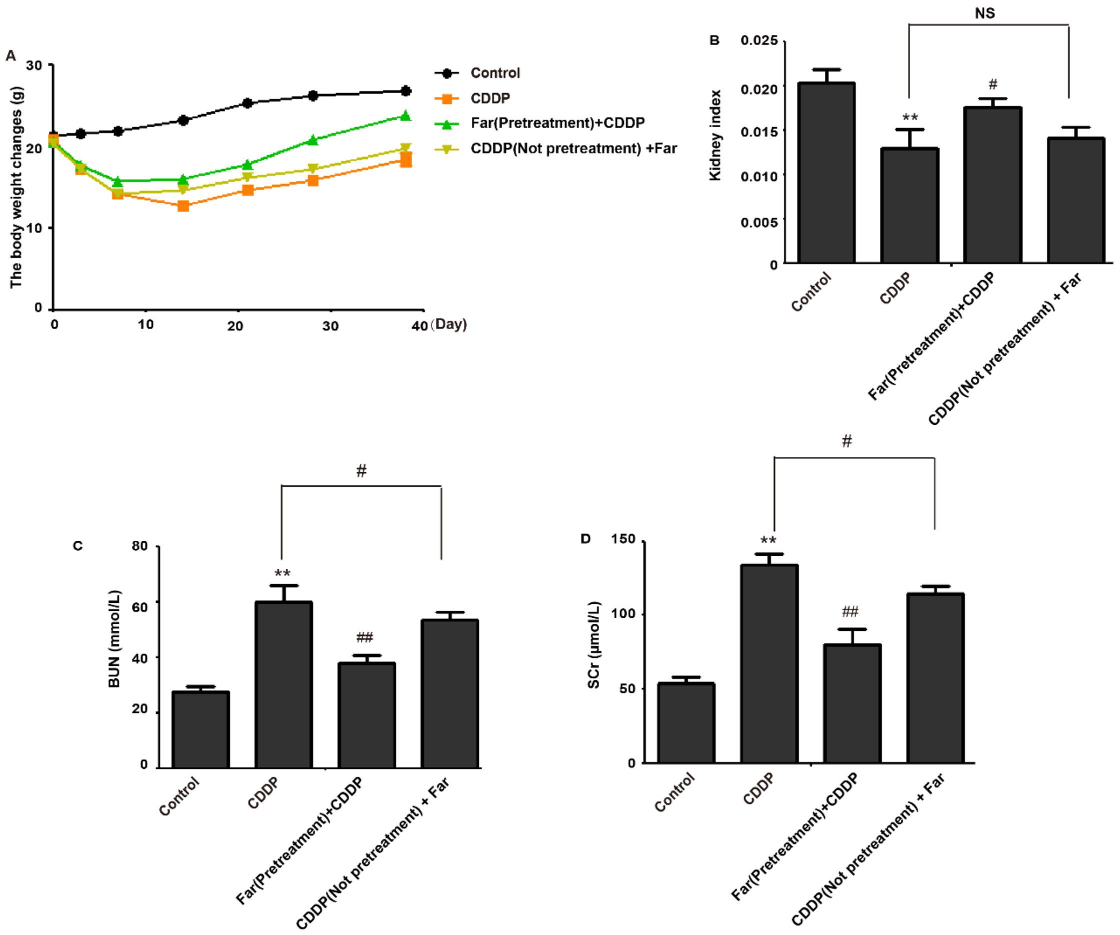


**SUPPLEMENTAL FIGURE S1. Comparison of CDDP-induced CKD mouse model pretreatment with farrerol and farrerol (not pretreatment) after cisplatin injection.** Mice were treated daily with farrerol (10 mg/kg/d) or vehicle beginning one hour after the first intraperitoneal cisplatin (10 mg/kg)) injection until five days after the second cisplatin injection. Subsequently, on the 31st day following the second CDDP administration, all mice were euthanized. Changes in weight (A) and kidney index (B) were evaluated. The collected whole blood was used to measure BUN (C) and SCr (D). The data are shown as the mean ± SEM ($n = 6$ in each group). All experiments were performed three times. *$p < 0.05$ and **$p < 0.01$ compared with the control group; #$p < 0.05$ and ##$p < 0.01$ compared with the CDDP group. NS, no specificity.
